# Integrative Taxonomic Study of the Purse Crab Genus *Persephona* Leach, 1817 (Brachyura: Leucosiidae): Combining Morphology and Molecular Data

**DOI:** 10.1371/journal.pone.0152627

**Published:** 2016-04-21

**Authors:** Tatiana Magalhães, Rafael Robles, Darryl L. Felder, Fernando L. Mantelatto

**Affiliations:** 1 Laboratório de Bioecologia e Sistemática de Crustáceos (LBSC), Departamento de Biologia, Faculdade de Filosofia, Ciências e Letras de Ribeirão Preto (FFCLRP), Universidade de São Paulo (USP), Ribeirão Preto, São Paulo, Brazil; 2 Department of Biology, University of Louisiana at Lafayette, Lafayette, Louisiana, United States of America; National Cheng-Kung University, TAIWAN

## Abstract

Marine crabs of the genus *Persephona* Leach, 1817 are restricted to American waters of the western Atlantic and eastern Pacific Oceans. Subfamilial assignment of this taxon has varied between authors and its species composition remain in question. We conducted a comparative study based on morphology and molecular phylogenetics for all ten recognized species of *Persephona*, along with *Iliacantha hancocki*. We tested whether *Persephona finneganae*, *P*. *lichtensteinii*, and *P*. *crinita* represent a single species as suggested by some authors; whether specimens identified as *P*. *punctata*, *P*. *mediterranea*, and *P*. *aquilonaris* warrant treatment as separate species; and whether *I*. *hancocki* should be regarded as a junior synonym of *P*. *subovata*. Diagnostic morphological characters (of the carapace, chelipeds, and third maxillipeds) were used along with gonopod (male first pleopod 1) features and live coloration. The 16S rRNA and the Cytochrome Oxidase I (COI) (DNA barcoding) mitochondrial genes were used as molecular markers. Both morphological and molecular analyses revealed that putative specimens of *P*. *crinita* from Brazil and those assigned to *P*. *finneganae* were no different from specimens presently assignable to *P*. *lichtensteinii*. *P*. *finneganae* is regarded as a junior synonym of *P*. *lichtensteinii*, and we apply *P*. *crinita* only to specimens we examined from the Gulf of Mexico. Specimens from Brazil previously reported as *P*. *crinita* are herewith concluded to represent *P*. *lichtensteinii*. Additionally, *P*. *townsendi* is a junior synonym of *P*. *orbicularis*, *Iliacantha hancocki* is concluded to be a junior synonym of *P*. *subovata*, while *P*. *aquilonaris* and *P*. *mediterranea* are found to represent separate species. On the basis of our revisions, eight species of *Persephona* are considered valid, and the reported distribution for *P*. *crinita* is restricted.

## Introduction

The family Leucosiidae Samouelle, 1819 encompasses the subfamilies Leucosiinae Samouelle, 1819, Cryptocneminae Stimpson, 1907, and Ebaliinae Stimpson, 1871. By one recent count, these three subfamilies contain a total of 63 genera and 464 species [1]. Usually referred as purse crabs, they are found most commonly on coastal sand bottoms, gravel, and muds, but some also occur in nearshore waters ranging to 400m in depth [2]. Systematics within the family remains in question at many levels, including the limits and definitions of the various subfamilies. The systematic position of *Persephona* Leach, 1817 is uncertain. It is currently included in the subfamily Ebaliinae Stimpson, 1871 [1, 2], but had previously been considered a member of the subfamily Philyrinae Rathbun, 1937 [3, 4]. Clearly, systematic relationships among the three subfamilies of Leucosiidae, Ebaliinae, Philyrinae, and Iliinae Stimpson, 1981, are not well resolved [1], although full treatment of this issue is beyond the scope of our present study.

According to current taxonomy, six species of *Persephona* are known to occur in the western Atlantic [*P*. *aquilonaris* Rathbun, 1933; *P*. *crinita* Rathbun, 1931; *P*. *finneganae* Rathbun, 1933; *P*. *lichtensteinii* Leach, 1817; *P*. *mediterranea* (Herbst, 1794), and *P*. *punctata* (Linnaeus, 1758)] and four in the eastern Pacific [*P*. *edwardsii* Bell, 1855; *P*. *orbicularis* Bell, 1855; *P*. *subovata* (Rathbun, 1893), and *P*. *townsendi* (Rathbun, 1893)] [1]). However, this number is somewhat uncertain given the questionable status of several species in the genus. Applying the Briggs and Bowen [5] scheme of American zoogeographic subdivisions, representative members of the genus *Persephona* are absent from only the eastern Pacific Juan Fernandez Province.

Within the genus, three species from the western Atlantic have had a confusing taxonomic history, owing to somewhat ambiguous morphological characters proposed for their separation. *Persephona crinita* Rathbun, 1931, described from off Mississippi in the northern Gulf of Mexico, has been reported widely from throughout the Gulf of Mexico to the Antilles, Venezuela, and Brazil. *Persephona lichtensteinii* Leach, 1817 was described from an unknown locality in the Americas and has been reported to occur in Venezuela, Suriname, French Guiana, and Brazil (Amapá to São Paulo). Finally, *Persephona finneganae* Rathbun, 1933, first reported from São Sebastião, São Paulo, Brazil, has been reported to occur in coastal waters of Haiti, Trinidad and Tobago, Venezuela, and French Guiana to Brazil (Amapá to Santa Catarina). The questionable separation of these three species has been based upon characters used in Rathbun’s [3] key, which indicates that *P*. *crinita* has nine carapace margin outgrowths or processes that she terms as short and tuberculiform “excrescences” ([3]: 152). By contrast, *P*. *finneganae* is said to have seven lateral and posterior spines plus two tubercles between the lateral and subhepatic margins, while *P*. *lichtensteinii* is defined as having seven lateral and posterior spines. Her accompanying diagnoses do little to elaborate on these supposed differences, and the character states become very difficult to judge across specimens of varied age and maturation. Holthuis [6] concluded that the tubercles between lateral and subhepatic spines, used to separate *P*. *finneganae* and *P*. *lichtensteinii*, were variable to the extent that they did not separate these two species, which he proposed to be synonymous. Moreover, Torres [7], in a morphological analysis of the specimens from Brazil and the United States, suggested that both *P*. *finneganae* and *P*. *crinita* from Brazil should be considered synonyms of *P*. *lichtensteinii*. However, the names of all three species remain in use [1, 8, 9].

Application of the species name *P*. *punctata* and names of its subspecies among western Atlantic populations also has a confusing history. The subspecies *P*. *punctata aquilonaris* Rathbun, 1933 was based upon specimens from St. Augustine on the Atlantic coast of Florida and defined to include specimens collected and identified as *P*. *punctata* by Stimpson [10] from Florida and South Carolina. Rathbun [3] further applied this subspecies name to early reports from South Carolina and Georgia (Gibbes [11]: therein referred to as *Guaia punctata*) and to specimens she identified from New Jersey, North Carolina, South Carolina, and Florida, ultimately delimiting her reported distribution to be from New Jersey to Texas. The morphological characters used to distinguish *P*. *punctata aquilonaris* from *P*. *punctata punctata* were presence of a granule instead of a broad tooth at the subhepatic angle (versus absent), a narrower and more produced front, and coarser granulation overall. She also made mention of possible differences in coloration, though as stated, these were less than definitive and not part of diagnoses.

The subspecies, *P*. *punctata punctata*, as defined by Rathbun [3], retained the original *P*. *punctata* and included *Cancer mediterraneus* Herbst, 1794, *Persephona latreillei* Leach, 1817, *Persephona lamarckii* Leach, 1817, and *Persephona guaia* Bell, 1855 as junior synonyms. She concluded that all of these were based upon western Atlantic material and defined the distribution of *P*. *punctata punctata* to be from the West Indies to Brazil. The separations and synonymies by Rathbun were re-examined by Guinot-Dumortier [12], who (on the basis of the male gonopod, carapace morphology, and coloration) proposed that *P*. *aquilonaris* warranted treatment as a separate species from *P*. *punctata*. She nonetheless concluded that the reddish-spotted *P*. *aquilonaris* from North America and the more uniformly colored *P*. *punctata* from the Antilles both ranged to southern Brazil. However, without specifically establishing a synonymy, Guinot-Dumortier ([12]: 433) noted that the published color pattern for the type of *P*. *mediterraneus* (Herbst, 1794), one species that Rathbun [3] had placed in the synonymy of *P*. *punctata punctata*, matched that of *P*. *aquilonaris* Rathbun, 1933 and thus might suggest that it should be the senior synonym. This resulted in subsequent confusion, as some authors applied the name *P*. *mediterranea* as the senior synonym [13–15] and others used *P*. *aquilonaris* for the same species [16]. In the most recent authoritative world account of brachyuran names both were listed as valid taxa [3].

The taxonomy of *Persephona* in the eastern Pacific also remains unresolved. Garth [17] proposed *P*. *townsendi* (Rathbun, 1893) to be a junior synonym of *P*. *orbicularis*. Garth questioned the distribution of *P*. *orbicularis*, excluding it from Chile (reported by Bell [18]). By doing so, the remaining distribution fell within the distribution of *P*. *townsendi*, which was from the Gulf of California to Ecuador. On this basis, he supported the proposed synonymy.

In his re-examination of the holotype of *Persephona subovata* (Rathbun, 1894), Hendrickx [19] noted its strong similarity to *Iliacantha hancocki* Rathbun, 1935, with these differing only in the size of peripheral carapace granules and the relative distance between posterior teeth. While he did not synonymize these species he noted they should at very least not be assigned to different genera, though the matter thus far has remained unresolved [3, 20, 21].

We herewith report on molecular phylogenetic and morphological analyses undertaken to test whether: 1) *Persephona finneganae*, *P*. *lichtensteinii*, and Brazilian populations of *P*. *crinita* represent a single taxon, as suggested by Holthuis [6] and Torres [7]; 2) specimens identified as *P*. *punctata*, *P*. *mediterranea*, and *P*. *aquilonaris* are separate species; 3) *Persephona townsendi* is a junior synonym of *P*. *orbicularis*; and 4) *Iliacantha hancocki* is a junior synonym of *P*. *subovata*.

## Material and Methods

### Sampling and collections

**Fig 1:**
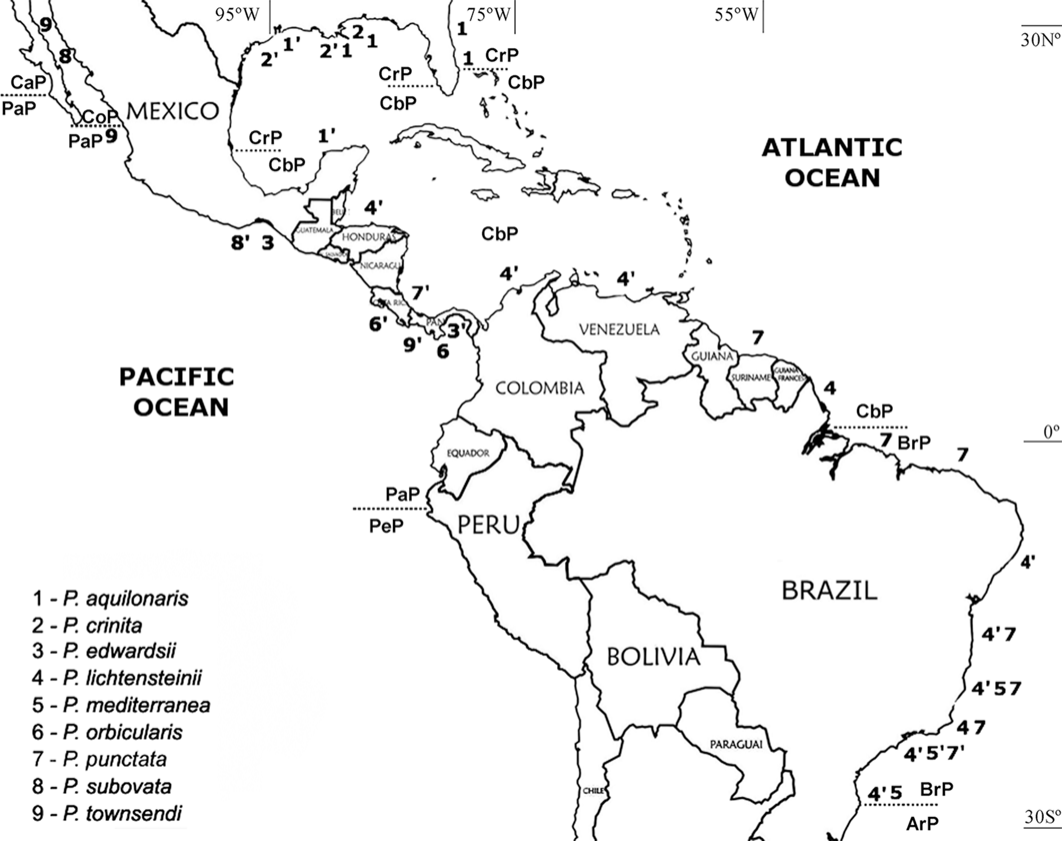
Collecting sites for specimens analyzed. An apostrophe the [‘] to right of number indicates that both morphological and molecular analyses were carried out on material from that site. Otherwise, only morphological analyses were conducted on that material. The dotted lines correspond to limits between zoogeographic provinces proposed by Briggs and Bowen [5]. Western Atlantic: CrP = Carolinian Province; CbP = Caribbean Province; BrP = Brazilian Province; ArP = Argentinian Province. Eastern Pacific: CaP = Californian Province; CoP = Cortezian Province; PaP = Panamanian Province; PeP = Peru-Chilean Province.

Specimens used in morphological analyses included materials of *Persephona* from the following regions: South Atlantic (Suriname, Amapá, Pará, Ceará, Alagoas, Bahia, Espírito Santo, São Paulo, and Santa Catarina); North Atlantic (Florida), Gulf of Mexico (United States and Mexico), and Caribbean Sea (Belize, Honduras, Costa Rica, Colombia, and Venezuela); Eastern Pacific: Mexico, Costa Rica, and Panama (Fig 1). Specimens representing these regions, along with two other species of Leucosiidae to serve as outgroups, were used in an accompanying molecular analysis (Table 1). Two other members of the subfamily Ebaliinae were selected as outgroups based on their phylogenetic proximity to the genus and availability of data.

**Table 1:** Crab specimens used in molecular analyses, with collection site, museum catalog number, and GenBank accession numbers. Collection abbreviations include CCDB (Coleção de Crustáceos do Departamento de Biologia, FFCLRP/USP); CNCR (Colección Nacional de Crustáceos, Instituto de Biología, UNAM); MNRJ (Museu Nacional da Universidade do Rio de Janeiro); MZUSP (Museu de Zoologia da Universidade de São Paulo); MZUCR (Museo de Zoología de la Universidad de Costa Rica); ULLZ (University of Louisiana at Lafayette Zoological Collection); USNM (National Museum of Natural History, Smithsonian Institution). AL-Alagoas; BA-Bahia; ES-Espírito Santo; SC-Santa Catarina; SP-São Paulo. Specimens in quotation marks were *a priori* identified as shown in the table.

Species	Collection site	Catalog no.	GenBank accession no.	
			COI	16S
*“Iliacantha hancocki”*	Costa Rica	CCDB 2834	JX102095	-
*Persephona crinita*	Texas, USA	ULLZ 1847	JX102076	JX102059
*Persephona crinita*	Louisiana, USA	ULLZ 11959	JX102077	JX102068
*Persephona crinita*	Mexico	CNCR 13954	KR818215	-
*“Persephona crinita”*	Caraguatatuba, SP, Brazil	CCDB 759	KR818216	-
*“Persephona crinita”*	Maceió, AL, Brazil	MZUSP 21087	JX102075	-
*“Persephona crinita”*	Ilhéus, BA, Brazil	MZUSP 21088	JX102074	JX102060
*Persephona edwardsii*	Panama	ULLZ 13932	JX102092	JX102070
*“Persephona finneganae”*	Cachoeira do Itapemirim, ES, Brazil	MNRJ 733	JX102078	-
*“Persephona finneganae”*	Venezuela	USNM 1155108	JX102079	JX102065
*Persephona lichtensteinii*	Ubatuba, SP, Brazil	CCDB 1430	-	JX102061
*Persephona lichtensteinii*	Ubatuba, SP, Brazil	CCDB 0023	JX102080	-
*Persephona lichtensteinii*	Ilhéus, BA, Brazil	MZUSP 21091	JX102081	-
*Persephona lichtensteinii*	Maceió, AL, Brazil	MZUSP 21096	JX102082	-
*Persephona lichtensteinii*	Bombinhas, SC, Brazil	MZUSP 13102	JX102083	-
*Persephona lichtensteinii*	Honduras	ULLZ 2002	JX102084	JX102069
*Persephona lichtensteinii*	Colombia	USNM 1072260	JX102085	JX102064
*Persephona mediterranea*	Ubatuba, SP, Brazil	CCDB 2662	JX102086	JX102067
*Persephona mediterranea*	Ubatuba, SP, Brazil	CCDB 1539	JX102087	-
*“Persephona mediterranea”*	Mexico	CNCR 17331	KR818217	-
*“Persephona mediterranea”*	Mexico	CNCR 14590	JX102088	-
*“Persephona mediterranea”*	Texas, USA	ULLZ 1840	JX102089	-
*Persephona orbicularis*	Costa Rica	CCDB 2939	JX102091	-
*Persephona puctata*	Ubatuba, SP, Brazil	CCDB 1581	JX102090	JX102063
*Persephona puctata*	Costa Rica	MZUCR 2470–4	KR818218	-
*Persephona subovata*	Mexico	CNCR 3269	JX102093	-
*“Persephona townsendi”*	Panama	ULLZ 13931	JX102094	JX102071
**Outgroups**				
*Ebalia cariosa*	Gulf of Mexico	ULLZ 6791	KR818220	KR81821
*Lithadia cadaverosa*	Louisiana, USA	ULLZ 5790	KR818219	KR81821

Intended for the molecular analyses, some fresh samples from the São Paulo state were collected complied with current applicable state and federal laws of Brazil (DIFAP/IBAMA/126/05; permanent license for collection of Zoological Material number 11777-MMA/IBAMA/SISBIO). All other specimens were obtained from museums and zoological collections as follow. Brazil: Coleção de Crustáceos do Departamento de Biologia (CCDB), Faculdade de Filosofia, Ciências e Letras de Ribeirão Preto (FFCLRP), Universidade de São Paulo (USP), Ribeirão Preto, São Paulo; Museu de Zoologia da Universidade de São Paulo (MZUSP) São Paulo, São Paulo; Museu Nacional da Universidade Federal do Rio de Janeiro (MNRJ), Rio de Janeiro, Rio de Janeiro; Museu de Zoologia da Universidade Estadual de Santa Cruz (MZUESC), Ilhéus, Bahia; Museu Oceanográfico da Universidade Federal do Pernambuco (MOUFPE), Recife, Pernambuco. Costa Rica: Museo de Zoología, Universidad de Costa Rica (MZUCR), San Jose, Costa Rica. Mexico: Colección Nacional de Crustáceos (CNCR), Instituto de Biología, Universidad Nacional Autónoma de Mexico (UNAM), Distrito Federal, Mexico. United States: National Museum of Natural History (USNM); Smithsonian Institution; Washington, DC; University of Florida Museum of Natural History (UFMNH), Gainesville, Florida; University of Louisiana at Lafayette Zoological Collection (ULLZ), Lafayette, Louisiana. Genetic voucher specimens from which tissue subsamples were distributed among the aforementioned collections (Table 1).

The indication of biogeographic provinces followed the classification of Briggs and Bowne [5]. Western Atlantic coast: Carolinian (Gulf of Mexico—from Cape Romano, Florida to Cape Rojo, Veracruz and Atlantic—from Cape Hatteras, North Carolina to Cape Canaveral, Florida), Caribbean (from Bermuda, Cape Canaveral, Florida, to the Amazon River), Brazilian (from Amazon River south to Santa Catarina) and Argentinian (from Santa Catarina to the Valdez Peninsula, Chubut). Eastern Pacific coast: Californian (from Los Angeles, California to Magdalena Bay, Baja California Sur), Cortezian (all the Gulf of California), Panamanian (from Magdalena Bay south to Gulf of Guayaquil), Galapagos (Galapagos Archipelago), Peru–Chilean (from the Gulf of Guayaquil to Taitao, Aysén) and Juan Fernández (Juan Fernández Islands).

### Molecular analyses

Molecular analyses were based on two mitochondrial genes, a fragment DNA of the 16S rRNA (16S) and the barcode region of the Cytochrome C Oxidase subunit I (COI). Both genes are widely used in phylogenetic studies of many invertebrates and decapod crustaceans [22–25]. More specifically for decapod crustaceans, both genes have been used in recent barcoding projects [26–28] and U.S. National Science Foundation Decapod Tree of Life phylogenetics studies [29–31] to delimit species boundaries and to clarify the evolutionary relationships among decapod crustaceans.

DNA extraction, amplification, and sequencing protocols followed Schubart *et al*. [32] with modifications according to Mantelatto *et al*. [33] and Robles *et al*. [34]. Total genomic DNA was extracted from muscle tissue of the chelipeds. The tissue was incubated for 48h in 600μL of lysis buffer at 55°C, with 200μL of proteinase K (PK); protein was separated by addition of 200μL of 7.5M ammonium acetate prior to centrifugation. DNA was precipitated by addition of 600μL of isopropanol cooled to the supernatant and then centrifuged; the resultant pellet was washed with ethanol 70%, centrifuged, dried and resuspended in 10–20μL TE buffer.

An approximately 550bp region of the 16S rDNA gene and 650bp region of the COI gene were amplified by polymerase chain reaction (thermal cycles: initial denaturing for 5min at 94–95°C; annealing for 35–40 cycles: 45sec at 95°C, 45sec at 42–48°C, 1min at 72°C; final extension of 5min at 72°C) with universal primers to invertebrates 16Sbr (5’-CCG GTC TGA ACT CAG ATC AC-3’) and 16Sar (5’-CGC CTG TTT ATC AAA AAC AT-3’) [35]; COH6 (5’-TAD ACT TCD GGR TGD CCA AAR AAY CA-3’) and COL6b (5’-ACA AAA TCA TAA AGA TAT YGG-3’) [36].

PCR products were purified using a SureClean Plus kit (following the vendor’s protocols) and sequenced using the ABI Big Dye^®^ Terminator Mix in an ABI Prism 3100 Genetic Analyzer^®^ following Applied Biosystems protocols. The sequences obtained were confirmed by sequencing both strands; consensus sequences were obtained using BioEdit version 7.0.5 [37] from the two complementary sequences. Consensus sequences of the 16S and COI genes were aligned using ClustalW [38] as implemented in BioEdit [37], with default parameters. The COI sequences were checked for the presence of stop codons. All sequences were submitted to GenBank.

#### Phylogenetic analyses

The concatenated analyses were conducted based on a total of 1091bp (606 for the COI and 485 for the 16S genes, excluding the primer regions). Alignment of both gene sequences was unambiguous and the ILD test showed no significant incongruence. After confirming that the two genes have the same evolutionary history, the best-fitting model for sequence evolution of the combined COI and 16S was determined by JModelTest 2.1.4 [39], selected by the AIC (Akaike information criterion) method. This information criterion indicated the TPM1uf+I+G as the best-fit model of DNA sequence evolution, accounting for invariable positions and unequal rates of substitutions under a gamma distribution, with the nucleotide frequencies: A = 0.3626, C = 0.1266, G = 0.1618, and T = 0.3490; rates of nucleotide substitution A-C = 1.0000, A-G = 94.1738, A-T = 7.0694, C-G = 7.0694, C-T = 94.1738, G-T = 1.0000; proportion of invariable sites I = 0.6530; and gamma shape = 0.8590.

The Bayesian analysis (BAY) was performed with MrBayes 3.2.2 [40] with the parameters obtained from JModeltest. The search was run with four chains for 20,000,000 generations with trees being sampled every 10,000 generations.

Trace plots were visually inspected to assess convergence, mixing, and stationarity in Tracer v1.4. [41]. Once the split frequency in each analysis was 1% (reached well before 2 million generations = 200 trees), we found the maximum clade credibility tree (MCCT) using TreeAnnotator v1.5.4 [42] from the remaining 1800 saved trees. We obtained a 50% majority rule consensus tree using the same 1800 trees. Bayesian posterior probability [43]; values > 70% were shown on the resulting MCCT.

Maximum Likelihood analysis (ML) was performed with RAxML 7.0.4 [44], as implemented in CIPRES (Cyberinfrastructure for Phylogenetic Research). The model of evolution was the GTR+G+I, which is the default model for RAxML. The internal consistency of the branches was evaluated by the bootstrap method [45], and we selected the option to automatically determine the number of bootstraps to be run in RAxML. A total of 150 bootstrap pseudoreplicates were run, and confidence values > 50% were shown on the resulting trees.

#### Genetic distance analyses

Genetic distance analyses were applied in previous systematic studies of crustaceans and other animals [46–50]. Genetic distance calculations were performed using MEGA 5 [51], and two distance matrices were calculated using uncorrected distances (*p*-distance), based on COI and 16S sequences. We did not calculate a distance matrix using a model of evolution since it has been shown that using *p*-distance avoid over-parametrizing and there is no need to use complex distances measures when studying closely related sequences [52, 53]. To help assess intraspecific and interspecific genetic distances, two frequency histograms were constructed with pairs of COI and 16S sequences.

### Morphological analyses

Analyses were conducted to evaluate morphological characters used most commonly for identifying members of *Persephona* in classical and current systematic literature (primarily features of carapace shape including the front, carapace armor or ornamentation, the third maxilliped, and the chelipeds). Additionally, the morphology of the first male gonopod (Go1) was comparatively reviewed [12, 54, 55, 56].

#### Scanning electron microscopy

For morphological study, Go1 were dissected from voucher specimens preserved in 70% alcohol and thereafter fixed in 3% glutaraldehyde, after which they were dehydrated in a graded ethanol series of 30%, 50%, 70%, 80%, 90%, 100% (30min changes each), dried in a critical point dryer with liquid CO_2_ in the EMS 850 (Electron Microscopy Sciences^®^), and sputter-coated with gold in Denton Vacuum Desk II coater. Examinations and micrograph exposures were conducted with a JSM 5410 (JEOL^®^) scanning electron microscope (SEM).

## Results

### Molecular analyses

#### Phylogenetic analyses

**Fig 2:**
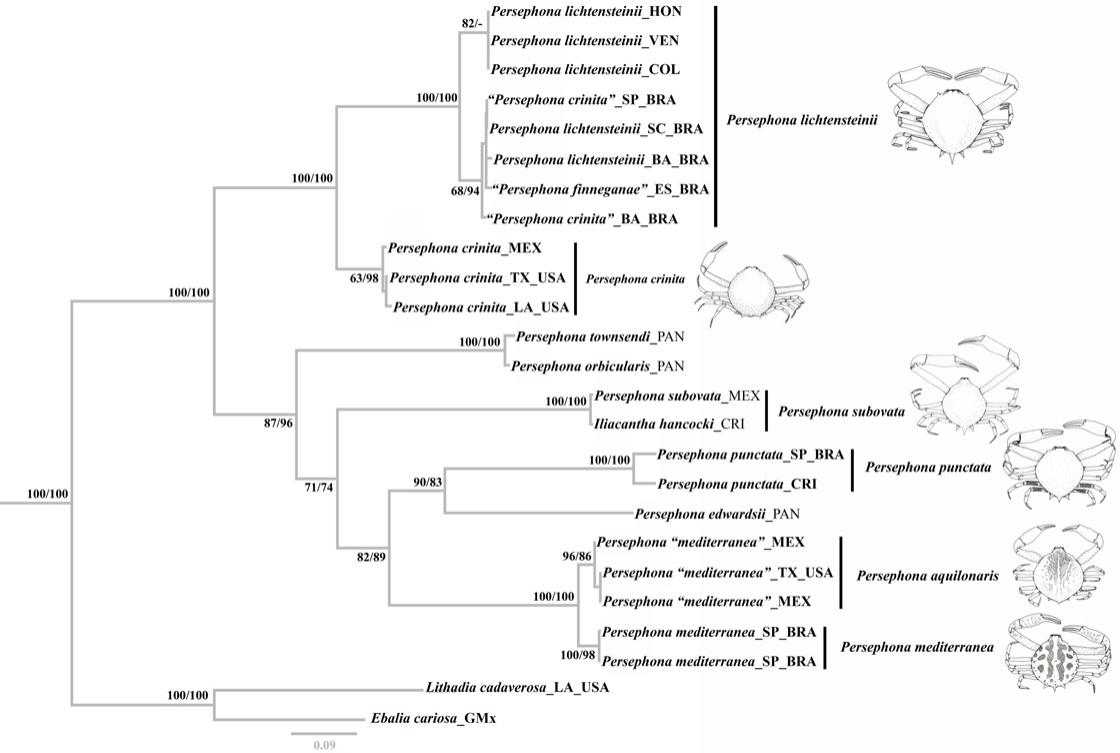
Bayesian tree for species of *Persephona*, and selected outgroups (*Ebalia cariosa* and *Lithadia cadaverosa*) based on the cytochrome oxidase I (COI) and large ribosomal subunit (16S) concatenated data set. Values represent bootstrap (ML) and Bayesian (BAY) posterior probabilities, expressed as percentages (ML/BAY). ML bootstrap values ≤ 50% are not shown; BAY posterior probabilities ≤70 are not shown either. AL-Alagoas; BA-Bahia; BRA-Brazil; COL-Colombia; CRI-Costa Rica; ES-Espírito Santo; GMx-Gulf of Mexico; HON-Honduras; LA-Louisiana; MEX-Mexico; PAN-Panama; SC-Santa Catarina; SP-São Paulo; VEN-Venezuela; TX-Texas. Atlantic localities are shown in bold. Specimens with quotation (“”) marks were *a priori* identified as shown in Table 1.

The ILD test showed no significant incongruence with the P = 0.874. Thus, we can consider the 16S and COI genes to have the same evolutionary history [57] and we could use the concatenated dataset for our phylogenetic analysis. Visual inspection of the two tree topologies obtained from the Bayesian and Maximum Likelihood analyses, based on all specimens analyzed, showed the topologies to be identical (Fig 2). All congeners of *Persephona*, along with *Iliacantha hancocki*, are joined in a highly supported clade in both the ML and BAY analyses. All congeners of *Persephona* along with *Iliacantha hancocki* are joined in a highly supported clade in both the ML and BAY analyses.

Analyzed specimens of “*P*. *mediterranea*” (here treated as *P*. *aquilonaris*) from Gulf of Mexico comprise a sister clade to *P*. *mediterranea* from Brazil. Together they form a highly supported sister group to the clade composed of *P*. *punctata* and *P*. *edwardsii*, with the latter clade exhibiting high support values in ML and BAY analysis.

*Persephona subovata* and *Iliacantha hancocki* cannot be distinguished from each other. The two specimens form a clade that is without support grouped with the clade encompassing *P*. *aquilonaris*, *P*. *mediterranea*, *P*. *punctata*, and *P*. *edwardsii* in ML and BAY analyses.

The species *P*. *orbicularis* and “*P*. *townsendi*” form a clade topologically placed as a sister to that containing *P*. *aquilonaris*, *P*. *mediterranea*, *P*. *punctata*, *P*. *edwardsii*, and *P*. *subovata*, with support in both the ML and BAY analyses. The topology also depicts *P*. *crinita* from Gulf of Mexico as the sister group of a clade composed by *P*. *lichtensteinii*, “*P*. *finneganae*”, and specimens of “*P*. *crinita*” from South America (treated here as *P*. *lichtensteinii*). Within this latter clade, Caribbean specimens form a clade that is sister to all *P*. *lichtensteinii* from Brazil with high support in ML analysis.

#### Genetic distance analyses

**Fig 3:**
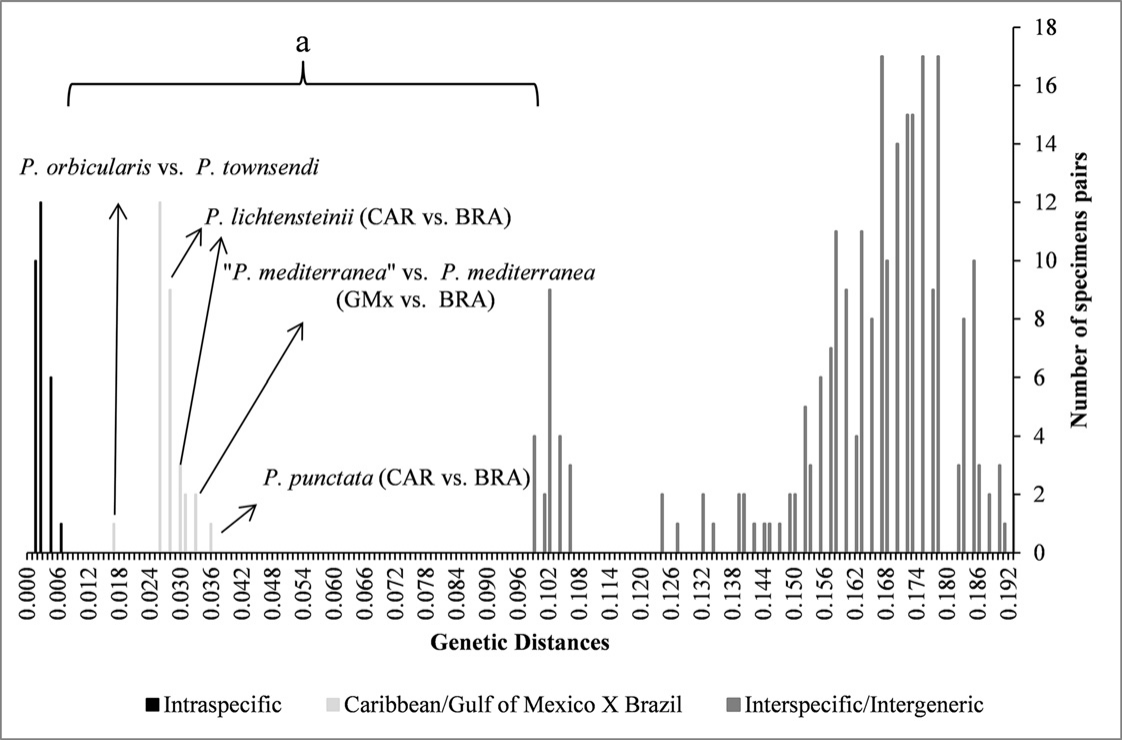
Histogram of *p*-distance for the cytochrome oxidase I (COI) gene. BRA = Brazil; CAR = Caribbean; GMx = Gulf of Mexico.

Genetic pairwise distance values based on COI show a clear separation among morphologically well-defined species (depicted as a wide gap, Fig 3A). The lower values, observed between individuals of the same species, ranged from 0 to 0.013 (mean ± SD = 0.007 ± 0.009) (e.g. specimens of *P*. *mediterranea* from Brazil), while the higher values, observed among organisms of different species (e.g. “*P*. *townsendi*” and *P*. *lichtensteinii*) or genera (e.g. “*P*. *townsendi*” and *E*. *cariosa*), ranged from 0.190 to 0.193 (mean ± SD = 0.192 ± 0.002) (Table 2; Fig 3).

**Table 2:** Pairwise genetic distance matrix of COI and 16S sequences among specimens of *Persephona*. Values above the diagonal are 16S and below the diagonal are COI genetic distances. BA-Bahia; BRA-Brazil; COL-Colombia; CRI-Costa Rica; ES-Espírito Santo; GMx-Gulf of Mexico; HON-Honduras; LA-Louisiana; MEX-Mexico; PAN-Panama; SC-Santa Catarina; SP-São Paulo; VEN-Venezuela; TX-Texas. Specimens with quotation (“”) marks were *a priori* identified as in Table 1.

Species	1	2	3	4	5	6	7	8	9	10	11	12	13	14	15	16	17	18	19	20	21	22	23	24	25
1_*Persephona_crinita*_LA_USA		0.000	-	0.030	-	-	0.028	0.028	0.028	-	-	-	0.051	-	0.051	-	0.048	-	-	0.044	-	0.055	-	0.067	0.074
2_*Persephona_crinita*_TX_USA	0.010		-	0.030	-	-	0.028	0.028	0.028	-	-	-	0.051	-	0.051	-	0.048	-	-	0.044	-	0.055	-	0.067	0.074
3_*Persephona_crinita*_MEX	0.013	0.003		-	-	-	-	-	-	-	-	-	-	-	-	-	-	-	-	-	-	-	-	-	-
4_"*Persephona_crinita*"_BA_BRA	0.102	0.102	0.099		-	-	0.002	0.002	0.007	-	-	-	0.057	-	0.057	-	0.051	-	-	0.053	-	0.057	-	0.076	0.083
5_"*Persephona_crinita*"_SP_BRA	0.104	0.104	0.101	0.005		-	-	-	-	-	-	-	-	-	-	-	-	-	-	-	-	-	-	-	-
6_"*Persephona_finneganae*"_ES_BRA	0.102	0.102	0.099	0.003	0.002		-	-	-	-	-	-	-	-	-	-	-	-	-	-	-	-	-	-	-
7_"*Persephona_finneganae*"_VEN	0.106	0.099	0.096	0.030	0.028	0.026		0.000	0.005	-	-	-	0.055	-	0.055	-	0.048	-	-	0.051	-	0.055	-	0.074	0.080
*8_Persephona_lichtensteinii*_COL	0.106	0.099	0.096	0.030	0.028	0.026	0.000		0.005	-	-	-	0.055	-	0.055	-	0.048	-	-	0.051	-	0.055	-	0.074	0.080
9_*Persephona_lichtensteinii*_HON	0.106	0.102	0.099	0.030	0.028	0.026	0.003	0.003		-	-	-	0.051	-	0.051	-	0.044	-	-	0.046	-	0.051	-	0.074	0.076
10_*Persephona_lichtensteinii*_BA_BRA	0.102	0.102	0.099	0.003	0.002	0.000	0.026	0.026	0.026		-	-	-	-	-	-	-	-	-	-	-	-	-	-	-
11_*Persephona_lichtensteinii*_SC_BRA	0.102	0.102	0.099	0.003	0.002	0.000	0.026	0.026	0.026	0.000		-	-	-	-	-	-	-	-	-	-	-	-	-	-
12_*Persephona_mediterranea*_SP_BRA	0.157	0.153	0.153	0.167	0.168	0.167	0.168	0.168	0.172	0.167	0.167		-	-	-	-	-	-	-	-	-	-	-	-	-
13_*Persephona_mediterranea*_SP_BRA	0.157	0.153	0.153	0.167	0.168	0.167	0.168	0.168	0.172	0.167	0.167	0.000		-	0.009	-	0.048	-	-	0.032	-	0.037	-	0.078	0.067
14_"*Persephona_mediterranea*"_MEX	0.160	0.157	0.157	0.178	0.180	0.178	0.183	0.183	0.186	0.178	0.178	0.033	0.033		-	-	-	-	-	-	-	-	-	-	-
15_"*Persephona_mediterranea*"_TX_USA	0.162	0.158	0.158	0.180	0.182	0.180	0.185	0.185	0.188	0.180	0.180	0.031	0.031	0.005		-	0.048	-	-	0.032	-	0.041	-	0.078	0.071
16_"*Persephona_mediterranea*"_MEX	0.158	0.155	0.155	0.177	0.178	0.177	0.182	0.182	0.185	0.177	0.177	0.031	0.031	0.008	0.007		-	-	-	-	-	-	-	-	-
17_*Persephona_punctata*_SP_BRA	0.150	0.152	0.152	0.173	0.175	0.173	0.175	0.175	0.173	0.173	0.173	0.140	0.140	0.142	0.139	0.142		-	-	0.039	-	0.048	-	0.080	0.069
18_*Persephona_punctata*_CRI	0.155	0.153	0.153	0.158	0.160	0.158	0.163	0.163	0.165	0.158	0.158	0.132	0.132	0.127	0.124	0.127	0.036		-	-	-	-	-	-	-
19_*Persephona_orbicularis*_CRI	0.149	0.155	0.155	0.178	0.177	0.178	0.183	0.183	0.185	0.178	0.178	0.172	0.172	0.170	0.172	0.172	0.165	0.168		-	-	-	-	-	-
20_*Persephona_edwardsii*_PAN	0.167	0.170	0.168	0.175	0.173	0.175	0.173	0.173	0.177	0.175	0.175	0.170	0.170	0.167	0.167	0.163	0.134	0.139	0.177		-	0.037	-	0.080	0.062
21_*Persephona_subovata*_MEX	0.163	0.165	0.162	0.172	0.170	0.172	0.178	0.178	0.178	0.172	0.172	0.167	0.167	0.155	0.152	0.158	0.155	0.144	0.173	0.167		-	-	-	-
22_"*Persephona_townsendi*"_PAN	0.155	0.162	0.162	0.185	0.183	0.185	0.190	0.190	0.188	0.185	0.185	0.168	0.168	0.173	0.175	0.175	0.165	0.172	0.017	0.183	0.183		-	0.083	0.080
23_"*Iliacantha_hancocki*"_CRI	0.163	0.165	0.162	0.175	0.173	0.175	0.178	0.178	0.178	0.175	0.175	0.163	0.163	0.152	0.149	0.155	0.152	0.147	0.177	0.163	0.003	0.186		-	-
24_*Lithadia_cadaverosa*_LA_USA	0.160	0.163	0.162	0.170	0.172	0.170	0.162	0.162	0.162	0.170	0.170	0.182	0.182	0.185	0.185	0.182	0.163	0.160	0.190	0.180	0.177	0.193	0.177		0.062
25_*Ebalia_cariosa*_GMx	0.150	0.155	0.155	0.160	0.162	0.160	0.158	0.158	0.158	0.160	0.160	0.170	0.170	0.175	0.173	0.173	0.152	0.145	0.191	0.165	0.157	0.193	0.157	0.124	

When genetic distances of specimens assigned to “*P*. *crinita”* and “*P*. *finneganae”* from the Brazilian Province were compared to those for specimens of *P*. *lichtensteinii* from the same province, values ranged from 0 to 0.003 (Table 2); these distance values fell within the range of intraspecific variation observed for other *Persephona* spp. (Fig 3). In contrast, when these same specimens of “*P*. *crinita*”, “*P*. *finneganae*” and *P*. *lichtensteinii* from the Brazilian Province were compared to specimens of *P*. *crinita* from the Gulf of Mexico (Carolinian Province), genetic distance values ranged from 0.102 to 0.104 (Fig 3; Table 2); these values fell within the range for interspecific distance.

The genetic distance value between *P*. *orbicularis* and “*P*. *townsendi*” (both specimens from the Panamanian Province) was 0.017, falling within the gap between intraspecific and interspecific genetic distance values (Fig 3A). The same was observed for specimens of *P*. *mediterranea* compared from three zoogeographic provinces. When two specimens from the Gulf of Mexico (one of “*P*. *mediterranea”* from the Carolinian Province and another from the Caribbean Province; see Fig 1) were compared to those from Brazil (two specimens from the Brazilian Province), values ranged from 0.031 to 0.033 (Fig 3), respectively. It is also noteworthy that when comparing the genetic distance between the two specimens of *P*. *mediterranea* from within the Brazilian zoogeographic province the value was 0.000, and when comparing the specimens of “*P*. *mediterranea*” from the Gulf of Mexico, the value ranged from 0.005 to 0.008 (Table 2).

Genetic distance values for compared specimens of *P*. *lichtensteinii* from two different zoogeographic provinces (Caribbean and Brazilian) fell in the interval between intraspecific and interspecific values at 0.026 (Fig 3). When all specimens of *P*. *lichtensteinii* from within a single province were compared, the value for genetic distance was 0.000 (Brazilian Province) and ranged from 0.000 to 0.003 (Caribbean Province) (Table 2; Fig 3).

Compared between two specimens of *P*. *punctata* from two different zoogeographic provinces (Caribbean and Brazilian), genetic distance values also fell between intraspecific and interspecific values (0.036; Fig 3). The observed genetic distance value between specimens of *P*. *subovata* and “*I*. *hancocki*” (both specimens from the Panamanian Province) was 0.003 (Table 2), within the values typically observed when comparing specimens within a single species of *Persephona*.

**Fig 4:**
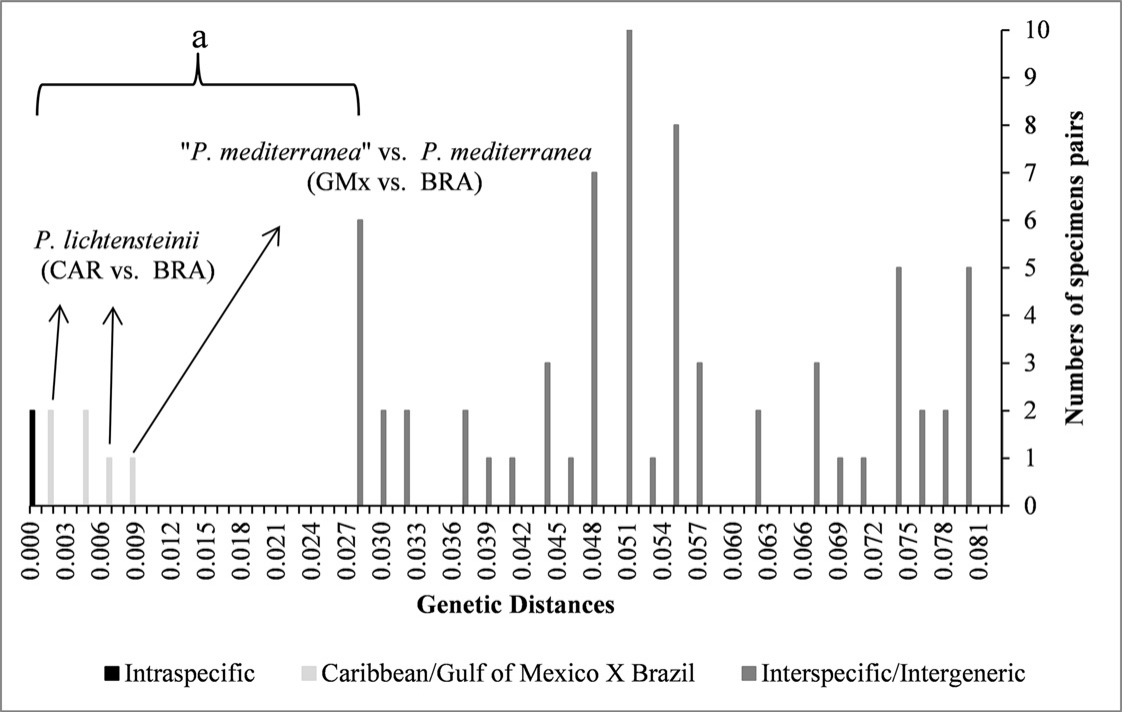
Histogram of *p*-distance for the large ribosomal subunit (16S). BRA = Brazil; CAR = Caribbean; GMx = Gulf of Mexico.

Genetic pairwise distance values based on the 16S gene, showed a clear separation among morphologically well-defined species (depicted as a wide gap; Fig 4A). The values observed between compared individuals of *P*. *crinita* from the Gulf of Mexico (Carolinian Province) and between compared individuals of *P*. *mediterranea* from Brazil (Brazilian Province) ranged from 0.000 to 0.010 (mean ± SD = 0.050 ±0.007). Values among compared specimens of different species (e.g. *P*. *crinita* from the Gulf of Mexico and *P*. *lichtensteinii; P*. *crinita* from the Gulf of Mexico and “*P*. *townsendi*”) ranged from 0.028 to 0.055 (mean ± SD = 0.042 ± 0.019) (Fig 4).

Pairwise distances within species of *Persephona*, based on the 16S gene, were obtained only for specimens of *P*. *crinita*, *P*. *lichtensteinii*, and *P*. *mediterranea*. Genetic distances between the specimens of “*P*. *mediterranea*” from the Gulf of Mexico (Carolinian Province) and one from Brazil (Brazilian Province) showed a genetic divergence of 0.010 (Fig 4, Table 2). These values fell within the gap that separates the intraspecific and the interspecific groups (Fig 4A).

In comparison of *P*. *lichtensteinii* and “*P*. *finneganae*” from Colombia and Venezuela, respectively, with *P*. *lichtensteinii* from Honduras (three specimens from the Caribbean Province) the value was 0.005 (Table 2). When comparing this same specimen from Honduras (Caribbean Province) to “*P*. *crinita*” from Brazil (Brazilian Province), the value was 0.007 (Fig 4; Table 2). However, even being in different provinces, comparing *P*. *lichtensteinii* and “*P*. *finneganae*” from Colombia and Venezuela, respectively, with *P*. *lichtensteinii* from Brazil the obtained value was 0.002. Thus genetic divergence values among specimens from the same zoogeographic province (Caribbean) were intermediate to values observed when comparing specimens from two different zoogeographic areas (Caribbean and Brazilian Provinces). These values also fell within the gap between what might be defined as an intraspecific rather than interspecific grouping (Fig 4A).

### Morphological analyses

**Fig 5:**
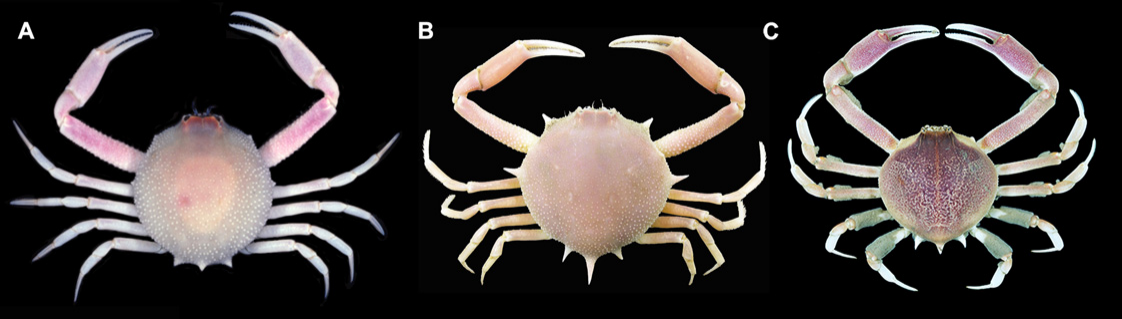
Selected western Atlantic species of *Persephona* differing in number and size of spines or granules, proportional width and length of the front, proportional widths and lengths of the cheliped articles and coloration of the carapace. Species include *P*. *crinita* ULLZ 1847, male, CW 17.3mm (A), *P*. *lichtensteinii* CCDB 5080, male, CW 16.0mm (B), and *P*. *punctata* CCDB 5169, male, CW 35.1mm (C). Image A by D. L. Felder; images B, C by R. C. Buranelli and L. A. G. Pileggi.

**Fig 6:**
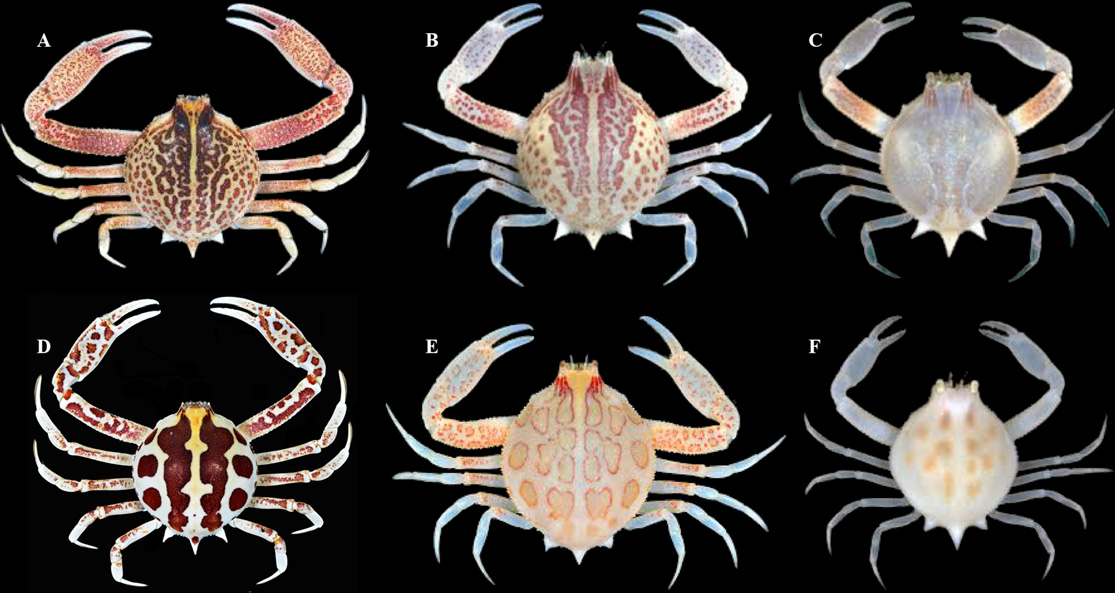
Ontogenetic changes in color patterning of *Persephona aquilonaris* (A–C) and *Persephona mediterranea* (D–F), both ranging from old (large) to young (small). Specimens include *P*. *aquilonaris*, ULLZ 1845, male, CW 26.2mm (A), ULLZ 4402, male, CW 15.0mm (B), ULLZ 8513, male, CW 7.9mm; *P*. *mediterranea*, MZUSP 33205, male, CW 32.8mm (D), UFMNH 6579, female, CW 19.0mm (E), ULLZ 15385, unsexed juvenile, CW 6.2mm. Image A–C, E, F by D. L. Felder; image D by R. C. Buranelli and L. A. G. Pileggi.

The morphological characters included number and size of spines or granules, proportion of width and length of the front, proportion of width and length of the chelipeds articles and coloration of carapace, all proving to be informative for identification of adults of *Persephona* (Fig 5). In addition, the carapace coloration pattern of two species, *P*. *aquilonaris* and *P*. *mediterranea* is informative even in juveniles (Fig 6).

#### Scanning electron microscopy

**Fig 7:**
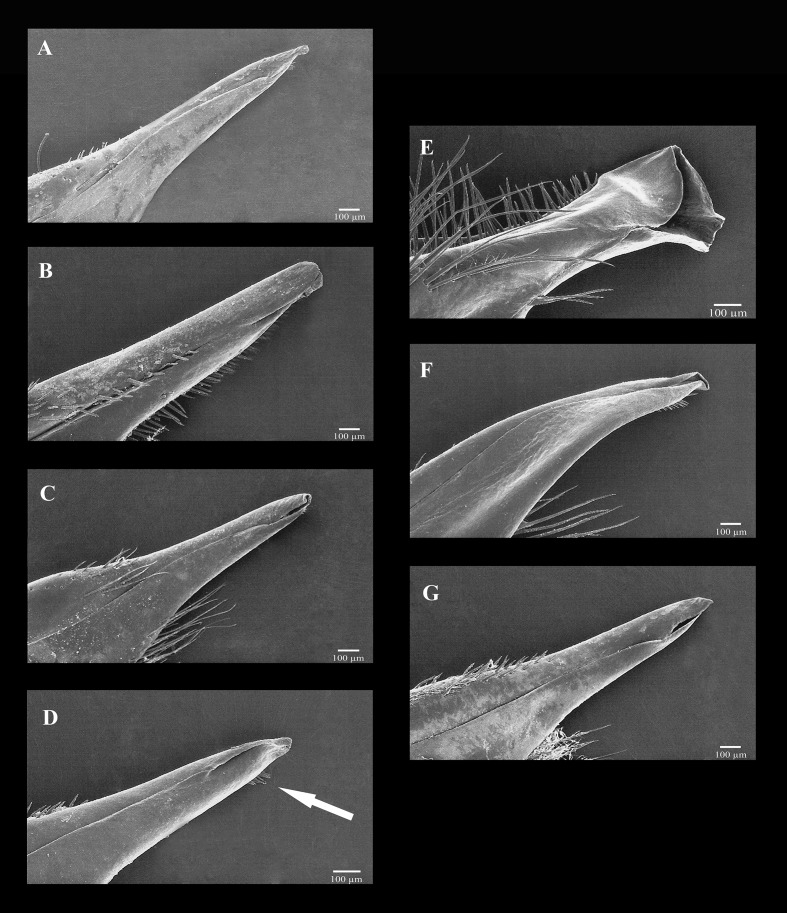
Scanning electron micrographs of the narrowed right Go1 tip, mesioventral face, for selected species of *Persephona*. Species included are *P*. *crinita* ULLZ 3902 (A), *P*. *edwardsii* CNCR 18997 (B), *P*. *lichtensteinii* CCDB 0011 (C), *P*. *orbicularis* CCDB 1638 (D), *P*. *punctata* CCDB 1581 (E), *P*. *subovata* CNCR 3269 (F), and *P*. *townsendi* CCDB 3024 (G). Arrow indicates subapical setae.

**Fig 8:**
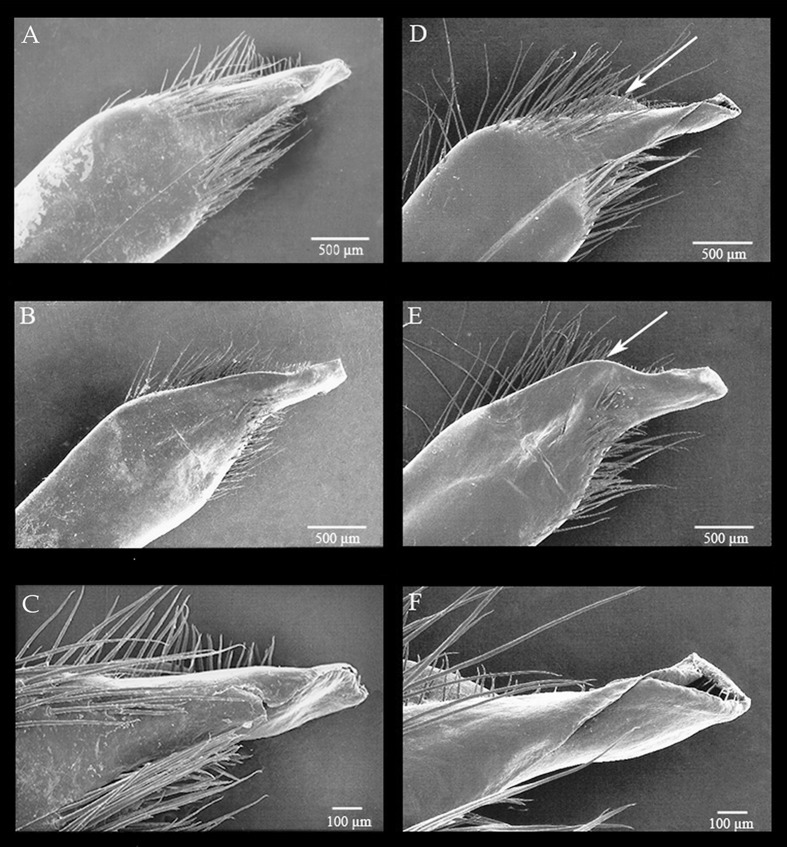
Scanning electron micrographs of the narrowed Go1 tip for *Persephona aquilonaris* ULLZ 1997 (A–C) and *P*. ***mediterranea* CCDB 2836 (D–F).** A, C, D, F, mesioventral face, right Go1; B, E, mesiodorsal face, left Go1. Arrows indicate prominent lobe. Collapsed opening of the Go1 of *P*. *aquilonaris* during preparation (A and C).

The morphology of the Go1 is species-specific (Figs 7A–7G; 8A–8C and 8D–8F), but with no clear evidence of importance for higher phylogenetic groupings. The principal differences were found in shape of the apex, presence or absence of subapical setae, and presence or absence of lobule on the mesiodistal margin (Fig 8). Furthermore, Go1 of *Persephona* is a stable character for males of different sizes and apparently does not change once the male minimum size has been reached.

## Discussion

### *Persephona crinita* Rathbun, 1931, *P*. *finneganae* Rathbun 1933, and *P*. *lichtensteinii* Leach, 1817

Our phylogenetic analyses showed clear separation between two well-supported clades of specimens identified as *P*. *crinita sensu lato*. One clade corresponds to specimens from the Gulf of Mexico (Carolinian province) and a second included specimens of “*P*. *finneganae*”, *P*. *lichtensteinii*, and those identified as “*P*. *crinita*” (Brazilian Province).

*Persephona crinita* is a species frequently reported in biodiversity studies of the Brazilian crustacean fauna [2, 8, 58, 59, 60]. However, specimens of “*P*. *crinita*” from Brazil usually are identified on the basis of a taxonomic key by Melo ([2]: 151), modified from the original one by Rathbun [61], and a drawing labelled as “*P*. *crinita*” that in fact depicts a color variant of *P*. *lichtensteinii* with poorly developed lateral spines. When analyzing specimens from the Gulf of Mexico, including the paratype of *P*. *crinita* (USNM 64254), it is evident that this species does not possess lateral spines but instead seven tubercles along the lateral margin, which are clearly depicted by Rathbun [61]. On the other hand, specimens from Brazil possess either seven spines, tubercles, or teeth that are of differing shapes and sizes, but these are noticeably more developed than are those of *P*. *crinita*. However, these marginal spines actually vary greatly in size, possibly due to wear resulting from the sand burrowing habits of these animals. To augment these sometimes variable spines as diagnostic characters, the morphology of the adult Go1 is clearly different in these two species. The opening in *P*. *crinita* (Fig 7A) is narrow and directed towards the mesiodorsal face while in *P*. *lichtensteinii* (Fig 7C) the opening is directed towards the mesioventral face.

In addition to her noting the possession of seven marginal spines, tubercles, or excrescences as already mentioned, Rathbun [61], [3] used the presence of tubercles between the sub-hepatic and the lateral tubercles to differentiate *P*. *crinita* (Fig 5A) from *P*. *lichtensteinii* (Fig 5B), a character also used to separate *P*. *finneganae* from *P*. *lichtensteinii*. However, this character is highly variable and can be evident or obscure, including on the type material [6, 7]. The posterior elongate spine on the carapace was also used by Rathbun [3, 62], to differentiate *P*. *finneganae* from *P*. *lichtensteinii*. It can be observed on individuals with or without tubercles, being present between the lateral and subhepatic spines, though it is not in itself a diagnostic character for *P*. *finneganae*. Another variable character utilized by Rathbun [62] to describe *P*. *finneganae* was the intestinal region of the carapace being partially defined by shallow furrows. This subjective character is present to varied degrees in all species of *Persephona* and is not of much value for diagnoses. Our examination of the carapace morphology and the Go1 of specimens assigned to *P*. *finneganae*, including the paratype (MZUSP 934) and the holotype (USNM 67989), support previous suggestions that *P*. *finneganae* is a junior synonym of *P*. *lichtensteinii* [6, 7]. Furthermore, our genetic distance analysis placed divergence values between these two species and materials from Brazil previously assigned to “*P*. *crinita*” within the range of intraspecific variation (even sharing some haplotypes), supporting the hypothesis that the three “species” are morphological variants of *P*. *lichtensteinii*. Thus, we contend that specimens currently assigned to *P*. *finneganae* and “*P*. *crinita*” from Brazil should now be referred to as *P*. *lichtensteinii*. Based upon our phylogenetic analyses, some conclusions on recent evolution of the group can be drawn, especially since both ML and BAY analysis also recovered the same topology. *Persephona crinita* (from the Carolinian Province) was recovered as a sister group of *P*. *lichtensteinii* (from the Caribbean and Brazilian Provinces) in all analyses and always with strong support. These species are in turn joined with strong support to the clade encompassing all other species of *Persephona*, including those from the Pacific. Internal support for this overall clade is strong, even while segregating Atlantic and Pacific species into separate subclades, suggesting that they diversified following closure of the Panamanian Isthmus. Dynamics contributing to diversification in the western Atlantic after emergence of the Isthmus may have included redirection of the North Equatorial Current in the northern hemisphere, this propelling the Gulf Stream through the Yucatan Strait, with this current becoming more intense than before the closure of the Isthmus [63]. In this scenario [63, 64] it is not difficult to envision barriers to dispersal, regional selective pressures, or isolation and sorting events that could result in divergence of a western Atlantic species pair from what was possibly a common evolutionary stock.

### *Persephona aquilonaris* Rathbun, 1933 and *P*. *mediterranea* (Herbst, 1794)

Specimens *a priori* identified as “*Persephona mediterranea*” from the Gulf of Mexico (Carolinian and Caribbean Provinces) were clustered in a well-supported clade together with specimens from Brazil (Brazilian Province) identified as *P*. *mediterranea*. However, it is clear from our phylogenetic analysis that there is some degree of genetic structure, which was also evident in the genetic divergence analysis. The genetic divergence distance based on COI was around 3%, which might or might not indicate variation expected at the population level [65]. Yet, in the analysis of 16S sequence data, the values for genetic distance were around 1%, which is above those observed for species separations among some other decapod crustaceans (i.e. species of mud crabs of the family Panopeidae; Schubart *et al*., [65]). Thus, we conclude that the analyzed specimens from the Gulf of Mexico (Carolinian and Caribbean Provinces) indeed represent a different species from those in South American waters. *Persephona aquilonaris* was described by Rathbun [62] for specimens from the northern hemisphere. However, the name was mostly abandoned following the work of Guinot-Dumortier [12]. When comparing specimens from Brazil to those from Louisiana, Texas, and eastern Florida (St. Augustine), including the type of *P*. *aquilonaris* (USMN 62057), we observed a few consistent morphological differences. The most definitive structural difference was found in morphology of the Go1, which exhibits a prominent lobe in *Persephona mediterranea* but not in *P*. *aquilonaris* (Fig 8A–8F). Gonopod morphology aside, their live coloration (which persists for short periods even after preservation) is also consistently different from other taxa. Although the color of the carapace for many specimens can be similar, the distribution pattern of the darker spots is usually distinct, especially in mature individuals. In adults of both species, the dark reddish to yellow-orange patterning of the carapace is somewhat bilaterally symmetrical, being subdivided by a mid-dorsal longitudinal track of light background color (Fig 6). However, in mature specimens of *P*. *aquilonaris*, the dorsal dark pattern consists of anastomosed small dark reddish brown spots, each usually with one to several light granules at its center, giving the spots a broadly ocellated appearance. The spots themselves tend to be loosely or continuously collected into longitudinally oblique tracts, separated by narrow lighter veins of background color, with the veins and lines of intersecting spots converging somewhat posteriorly and the pattern broken into isolated small spots posterolaterally (Fig 6A–6C; see also Williams [66]: 150, fig. 127, as *P*. *punctata aquilonaris*; Williams [4]: 288, fig. 223 as *P*. *mediterranea*). By contrast, in *P*. *mediterranea*, the darker components of the dorsal pattern usually include 7–14 large reddish brown to light orange or tan spots, each usually margined by a narrow darker line encircling multiple granules, giving large spots a narrowly ocellated appearance. These robust spots of somewhat varied darkness are separated by broad lighter veins of lighter background color, with the veins and rows of the large spots not obviously converging posteriorly nor broken into a pattern of isolated small spots posterolaterally (Fig 6D–6F; see also Herbst [67]: 150, tab. 37, fig. 2 as *Cancer mediterraneus*; Guinot-Dumortier [12]: 432, fig. 9 as *P*. *aquilonaris*; Melo [2]:153 unnumbered figure). In both species, subadult and immature specimens are less conspicuously patterned and very small juveniles may almost totally lack most dark pigmentation of the carapace.

On the basis of our molecular phylogenetic analysis, genetic distance analysis, morphological comparisons of the Go1, and comparisons of adult color patterns, we conclude that *P*. *aquilonaris* and *P*. *mediterranea* should be treated as separate species. In general, *P*. *aquilonaris* applies to most populations distributed from New Jersey to Texas, with *P*. *mediterranea* applying to primarily those populations known to be distributed from the Antilles and Caribbean through southern Brazil, albeit with a slight overlap of the two species in tropical waters of the eastern Gulf of Mexico. While we separate *P*. *aquilonaris* and *P*. *mediterranea*, our phylogenetic analyses grouped them as sister species in a clade with high support values in ML and BAY analyses. We regard them as northern and southern counterparts that likely diverged from common stock over a long history of glacial advance and retreat following closure of the Panamanian Isthmus [63].

### *Persephona punctata* (Linnaeus, 1758) and *P*. *edwardsii* Bell, 1855

It is clear in our analyses that the Atlantic *P*. *punctata* deserves species rank, independent of its former subspecies, *P*. *aquilonaris*, but less expected that it resolves as a sister species of the Pacific congener, *P*. *edwardsii*, in both analyses with high support for ML and BAY analyses. The interspecific genetic distance was about 17% for COI and around 3% for 16S. Furthermore, the carapace armature and Go1 morphology (Fig 7B and 7E) are considerably different. Nonetheless, these two species are the members of the genus in which the carpus and merus of the cheliped share the character of bearing tufts of setae on the inner margins. This appears to represent yet another example among marine decapod crustaceans of a species pair diverging from a common ancestor after gene flow was interrupted by closing of the Isthmus of Panama (~3.5 mybp [68]). A growing number of such amphi-American or trans-isthmian species pairs are now underpinned by molecular phylogenetic analyses that provide a measure of genetic divergence since separation [35, 68, 69, 70]. It is noteworthy that lacking modern genetic tools, Rathbun [3] had already regarded *P*. *subovata* rather than *P*. *edwardsii* as the Pacific analog of the Atlantic *P*. *punctata*.

### *Persephona orbicularis* Bell, 1855 and *P*. *townsendi* (Rathbun, 1893)

*Persephona orbicularis* was recovered in the same clade as “*P*. *townsend*i” with high support in ML and BAY analyses. Furthermore, the genetic divergence value in COI was 1.7%, which is between values corresponding to intraspecific and interspecific genetic distance. The specimens are also very similar in their morphological characteristics. Rathbun [3] distinguished these species based on the shape and size of the spines in the sub-hepatic margin, which were reported to be long and acute in *P*. *townsendi* but shorter and dentiform in *P*. *orbicularis*. However, as previously noted, such characters are unreliable because these crabs burrowing habits may cause wear [71], [72]. It has been reported also that these crabs show sexual dimorphism in that spines are more developed in males of *P*. *orbicularis* than in females (Boone [73], pl. 11, figs. A, B). We were also able to confirm that these spines are more developed on the type male of *P*. *townsendi* (USNM 17382) than in the female type of *P*. *orbicularis* (USNM 17382). Those variable characters were, however, used by Rathbun [3] to separate these two species. In addition to our finding little genetic difference between these putative species, we found their Go1 structures to be remarkably similar (Fig 7D and 7G), without features to separate them. Thus, based on the molecular and morphological evidence, and their overlapping distributions, the two species are synonymized. As *P*. *orbicularis* is the older name available, *P*. *townsendi* becomes a junior subjective synonym of *P*. *orbicularis*.

### *Persephona subovata* (Rathbun, 1893) and *Iliacantha hancocki* Rathbun, 1935

In our analysis, the separation of *P*. *subovata* and “*I*. *hancocki*” cannot be supported. The genetic divergence value obtained with COI was only 0.3%, clearly within the range of intraspecific genetic variation, placing “*I*. *hancocki*” and equivalent to other existing species in the genus *Persephona*.

Previous study of the morphological similarities between these species revealed no significant differences upon which to base separations and encouraged further morphological studies with the inclusion of type materials [19]. From our studies of specimens representing “*I*. *hancocki*” and *P*. *subovata* from Costa Rica and Panama, along with type materials of both species (*I*. *hancocki*, USNM 69260; *Myra subovata*, USNM 17385), we could find no consistently definitive morphological characters to use for distinction. The characters used by Rathbun [74] to establish *I*. *hancocki* are all deemed to be variations broadly representative of *P subovata*. For example, the presence of a definite line of granules on the lateral margins, described to occur in *P*. *subovata* but not *I*. *hancocki*, is in fact present in the holotype of the later. Also the holotypes of *I*. *hancocki* as well as *P*. *subovata* exhibit a series of granules on the subhepatic region instead of a “rudimentary tooth” as described by Rathbun [74] exclusively for *I*. *hancocki*. Rathbun [3] noted that the chelipeds of *I*. *hancocki* are long, but much less slender than the others allied species. The inconsistency of the morphological characters separating these two species was also noticed by Hendrickx [19] who found differences only in “the size of the granules on the periphery of the carapace and the relative size of the posterior teeth”. These two characters also appear to be variable and dependent on the maturity of the specimens, based on different specimens analyzed in the present study. Effects of ontogeny on the distance between the posterior teeth was also found to be variable in other species of *Persephona* (i.e. *P*. *lichtensteinii*) and is thus generally of questionable value. Thus, based on the molecular and morphological evidence, the two species must be synonymized. As *P*. *subovata* is the older name available, *I*. *hancocki* becomes a junior subjective synonym of *P*. *subovata*.

The above synonymization of *I*. *hancocki* does not affect the taxonomic status of the genus *Iliacantha*. There are five more species within *Iliacantha* for which we do not have genetic data to assess the validities of the two genera. However, we are able to separate both genera based on morphology of the cheliped alone.

The original characters described by Stimpson [75] to separate *Iliacantha* are mostly useless, i.e. the three spines at the posterior extremity of the carapace, used to diagnose *Iliacantha*, can be also found on *Persephona*; the extremities of the pterygostomian channels projecting considerably beyond the orbits and the fused abdominal segments 3–5 and 4–6, for males and females respectively, are also present on all members of *Persephona* [76]. The characters of the cheliped that can separate the two genera are as follow: *Iliacantha* has elongated chelipeds, slender fingers with similar thickness throughout its length. On the other hand, *Persephona* has the chelipeds rather massive, with large fingers whose thickness decreases from the base to the apex. Additionally, specimens of *Iliacantha* have a peculiar orientation of the hands, which are twisted in the way that the fingers open in a vertical, instead of a horizontal plane. In what refer to those characters, *Iliacantha hancocki* resembles more members of *Persephona* than those of *Iliacantha*.

## Conclusions

Based on molecular and morphological analyses, we propose substantial modifications to the current taxonomy of the genus *Persephona*. We restrict the distribution of *P*. *crinita* to the Gulf of Mexico; we synonymize *P*. *finneganae* under *P*. *lichtensteinii*; we confirm that *P*. *mediterranea* and *P*. *aquilonaris* are both valid species, the first occurring in part of southern Florida, the Caribbean and South America, the second restricted to the Gulf of Mexico and eastern North America. We also show that *P*. *townsendi* is a junior synonymy of *P*. *orbicularis*, and that *Iliacantha hancocki* is a junior synonym of *P*. *subovata*. Following this revision, there are eight valid species of *Persephona*.

## Systematic Revisions

Genus ***Persephona*** Leach, 1817

= *Persephona* Leach, 1817 [76] (type species *Persephona latreillei* Leach, 1817 [76], gender feminine)

= *Guaia* H. Milne-Edwards, 1837 [77] (type species *Cancer punctatus* Linnaeus, 1758 [78], gender feminine)

**Included species:** *P*. *aquilonaris* Rathbun, 1933, *P*. *crinita* Rathbun, 1931, *P*. *edwardsii* Bell, 1855, *P*. *finneganae* Rathbun, 1933, *P*. *lichtensteinii* Leach, 1817; *P*. *mediterranea* (Herbst, 1794), *P*. *orbicularis* Bell, 1855, *P*. *punctata* (Linnaeus, 1758), *P*. *subovata* (Rathbun, 1893), and *P*. *townsendi* (Rathbun, 1893).

***Persephona aquilonaris*** Rathbun, 1933

Figs 6A, 8A–8C

*Guaia punctata*.―Gibbes, 1850: 185 [11].

*Persephona punctata*.―Stimpson, 1859: 70 [10].―Hay and Shore, 1918: 423, pl. 32, fig. 9 [79].

*Persephona punctata aquilonaris* Rathbun, 1933: 184 [62].―Rathbun, 1937: 154, pl. 42, figs. 6–7 [3].―Behre, 1950: 23 [80].―Hildebrand, 1954: 276 [81].―Wass, 1955: 154 [82].―Williams, 1965: 150, fig. 127 [66].―Overstreet and Heard, 1978: 132 [83].

*Persephona aquilonaris*.―Guinot-Dumortier, 1959: 429–432 [part, New Jersey to Texas specimens only] [12].―Tabb and Manning, 1961: 600 [84].―Rouse, 1970: 24, fig. 65 [85].―Felder, 1973: 4, 40, 42, pl. 5, fig. 4 [86].―Taissoun, 1986–88: 132–133 [87].―Ng *et al*., 2008: 92 [1].―Ortíz and Olcha, 2011: 51 [88].

*Persephone punctata* [sic].―Kingsley, 1878: 324–325 [89].―Cerame-Vivas and Gray, 1966: 263 [90].

*Persephona mediterranea*.―Abele, 1970: 62–63 [part, New Jersey to Campeche specimens only] [91].―Powers, 1977: 39 [16].―Williams, 1984: 288, fig. 223 [4].―Abele and Kim, 1986: 42, 481, 486–487, fig. i [13].―Raz-Guzman and Sánchez, 1992: 30, pl. I,1 [14].

*Persephona punctata acquilonaris* [sic].―Collins *et al*., 2009: 28 [92].

Material examined.―Holotype.―Saint Augustine, Florida, United States, id: L. W. Schmitt, coll: R. Ranson, 1 ♂ of *Persephona aquilonaris* (CW 43.0mm), USNM 62057. Other specimens.―United States: Texas ULLZ 1514 (1 ♂); ULLZ 1997 (1 ♂ reported as *P*. *mediterranea*). Mississippi ULLZ 9407 (1 ♂, 1 juvenile). Louisiana ULLZ 3382 (1 ♀ ov), 3379 (1 ♂). Florida USNM 2092 (1 ♂, 1 ♀ov); ULLZ 10158 (1 juvenile reported as *P*. *mediterranea*). Mexico: CNCR 14590 (1 ♂ reported as *P*. *mediterranea*), 17331 (1 ♂ reported as *P*. *mediterranea*).

Revised diagnosis.―Carapace with three marginal spines on posterior margin; adult background carapace color light beige to pale yellow, typically marked by strong dark reddish brown patterning of anastomosed spots distributed symmetrically to each side of light unspotted median line, dark spots separated by narrow lighter veins of background color, veins and lines of intersecting spots converging somewhat posteriorly, pattern broken into isolated small spots posterolaterally; front prominent, width usually two times length. Cheliped propodus usually more than three times longer than wide, carpus and distal part of merus, without tufts of setae on inner margins. Go1 distal opening large, directed towards mesioventral face; apex simple, without prominent lobe on mesiodistal margin, without subapical setae (Fig 8A–8C).

Distribution.―Western Atlantic: New Jersey to Gulf of Mexico.

Remarks.―See more discussion on remarks of *P*. *mediterranea*.

***Persephona crinita*** Rathbun, 1931

Figs 5A and 7A

*Persephona crinita* Rathbun, 1931: 128.―Rathbun 1937: 163–164, pl. 43, figs. 2–3, pl. 44, figs. 1–3 [3].―Wass, 1955: 154 [82].―Compton, 1962: 11 [93].―Felder, 1973: 39–40, pl. 5, fig. 3 [86].―Powers, 1977: 39 [part., Florida to Texas specimens only] [16].―Rodriguez, 1980: 256 [94].―Abele and Kim, 1986: 42, 481, 486–487, figs. e–h [13].―Vázquez-Bader and Gracia, 1994: 74 [95].―Melo, 1996: 151 (figure and map.) [part, Gulf of Mexico specimens only] [2].―Camp *et al*., 1998: 146 [96].―Álvarez *et al*., 1999: 10 [97].―Boschi, 2000: 112 [98].―Ng *et al*., 2008: 92 [1].―Felder and Camp, 2009: 1075 [15].―Ruiz *et al*., 2013: 282 [99].

Material examined.―Paratype.―Horn Island Pass, Mississippi, United States, 20 August 1930, id: M. J. Rathbun, coll: S. Springer, 1 ♂ of *Persephona crinita* (CW 19.4mm), 1 ♀ of *Persephona crinita* (CW 23.8mm) and 1 juvenile of *Persephona crinita* (CW 16.2mm), USNM 64254. Other specimens.―United States: Texas ULLZ 1995 (1 ♂), 8485 (1 juvenile), 1847 (1 juvenile); USNM 1155109 (1 ♂). Mississippi ULLZ 879 (2 ♂, 1 ♀, 1 juvenile). Louisiana ULLZ 3343 (1 ♀ov), 3349 (1 juvenile), 3350 (1 juvenile), 3902 (1 ♂), 11942 (1 ♀), 11956 (1 juvenile), 11959 (1 ♂). Mexico: CNCR 13954 (1 ♂).

Revised diagnosis.―Carapace with seven marginal spines or tubercles, three short spines or tubercles on posterior margin, two tubercles on lateral margin, two tubercles at subhepatic margin; adult background carapace color pale rose pink to bluish off-white, sometimes with poorly defined slightly darker rose area centered on and dominating most of dorsal surface, often with diffuse pale brown to orange near frontal margin, without symmetrical dark patterning of lines or spots; front slightly produced, width usually more than four times length. Cheliped propodus length usually more than four times width, carpus and distal part of merus without tufts of setae on inner margins. Go1 distal opening narrow, directed towards mesiodorsal face; apex simple, without prominent lobe on mesiodistal margin, with subapical setae (Fig 7A).

Distribution.―Western Atlantic: Gulf of Mexico.

Remarks.―The type of *Persephona crinita* was requested from the USNM where it is now kept (USNM63739). Unfortunately, the type remains on loan to another colleague and we were unable to examine it. However, using the original description of Rathbun [61], the figures of Rathbun ([3], pl. 43, figs. 2, 3; pl. 44, figs. 1–3) and the analyses of the paratype (USNM 64254), we are sure about the identification of *P*. *crinita*.

***Persephona edwardsii*** Bell, 1855

Fig 7B

*Persephona edwardsii* Bell, 1855: 294, pl. 31, fig. 8 [18].―Stimpson, 1859: 70 [10].―Rathbun, 1937: 154, pl. 45, figs. 3–4 [3].―Garth, 1946: 358 [100].―Garth, 1960: 121 [101].―Garth, 1966: 9 [102].―Buitendijk, 1950: 270 [103].―del Solar *et al*., 1970: 26 [104].―Sosa-Hernández *et al*., 1980: 17, pl. 3 [105].―DiMauro, 1982: 174 [106]

Correa-Sandoval, 1991: 12 [107].―Hendrickx, 1993: 8 [108].―Hendrickx, 1995: 129 [109].―Hendrickx, 1997: 146 [19].―Boschi, 2000: 112 [98].―Ng *et al*., 2008: 92 [1].―Vargas-Castillo, 2008: 108 [110].―Moscoso, 2012: 19 [111].

Material examined.―Photograph of syntype (by M. Carnall).―Galapagos Islands, Ecuador, 1 ♀ of *Persephona edwardsii* (CW 33.0mm), OUMNH 13777. Other specimens.―Mexico: Chiapas CNCR 18997 (2 ♂). Panama: ULLZ 13932 (1 juvenile).

Revised diagnosis.―Carapace with three marginal spines or tubercles, three long spines on posterior margin; adult carapace background color pale rose pink to bluish off-white, sometimes with poorly defined slightly darker rose area centered on and dominating most of dorsal surface, without symmetrical dark patterning of lines or spots; front slightly produced, width usually three times length. Cheliped propodus length usually more than three times width, carpus and distal part of merus with tufts of setae on inner margins. Go1 distal opening narrow, directed forward; apex simple, without prominent lobe on mesiodistal margin, with a row of setae from the base to apex (Fig 7B).

Distribution.―Pacific Oriental: Mexico (Punta Piaxtla, Sinaloa to Chiapas), Panama, Ecuador and Peru.

Remarks.―The type locality of *P*. *edwardsii* was considered to be the Galapagos Islands [18]. However, Garth [112] clarified that *P*. *edwardsii* was collected by Hugh Cuming, and only later said to have originated from the Galapagos Islands. Since this and other of Bell’s reported collections could not subsequently be found in the Galapagos Islands, Garth removed *P*. *edwardsii* from the list of known Galapagos brachyuran crabs. Thus, the type locality of *P*. *edwardsii* remains in question.

***Persephona lichtensteinii*** Leach, 1817

Figs 5B and 7C

*Persephona lichtensteinii* Leach, 1817: 23 [76].―Bell, 1855: 293–294, tab. XXXI, fig. 6 [18].―Rathbun, 1937: 152, 163, pl. 45, figs. 1–2 [3].―Holthuis, 1959: 181–182 [6].―Rodriguez, 1980: 255 [94].―Coelho and Ramos, 1972: 183 [113].―Gomes-Corrêa and Silva-Brum, 1980: 61 [114].―Coelho *et al*., 1983: 154 [115].―Takeda, 1983: 115 (unnumbered figure) [116].―Coelho *et al*., 1986: 74 [117].―Melo, 1996: 152 (unnumbered figure, map) [2].―Torres, 1998: 106, fig. 27 [7].―Rieger *et al*., 1999: 193, fig. 1 [118].―Boschi, 2000: 112 [98].―Mantelatto and Fransozo, 2000: 702–703 [8].―Mantelatto *et al*., 2003: 212 [119].―Bertini *et al*., 2004: 2190, 2192, 2204 [120].―Almeida *et al*., 2008: 32 [121].―Coelho *et al*., 2008: 15 [122].―Ng *et al*., 2008: 93[1].―Carvalho *et al*., 2010: 109–113 [123].―Hirose *et al*., 2012: 17–30 [9].―Almeida *et al*., 2013: 1581–1589 [60].―Furlan *et al*., 2013: 1349, 1351 [124].

*Persephone lichtensteinii* [sic].―Moreira, 1903: 120 [125].―Luederwaldt, 1919: 435 [126].

*Persephona lichtensteini* [sic].―Finnegan, 1931: 614–615, text-fig. 2 [127].―Werding and Müller, 1989: 410, fig. 5 [128].

*Persephona finneganae* Rathbun, 1933: 184 [62].―Rathbun, 1937: 152, 161, 163, pl. 42, fig. 37 [3].―Guinot-Dumortier, 1959: 434, fig. 8 a–c [12].―Coelho and Ramos, 1972: 183 [113].―Gomes-Corrêa and Silva-Brum, 1980: 61 [114].―Coelho *et al*., 1983: 154 [115].―Coelho *et al*., 1986: 74 [117].―Taissoun, 1986–88: 128, 129 (fig.) [87].―Ng *et al*., 2008: 93[1].

*Persephona crinita*.―Coelho and Ramos, 1972: 183 [113].―Coelho and Torres, 1980: 72 [129].―Coelho *et al*., 1986: 74 [117].―Melo, 1996: 151 (figure and map) [part, Caribbean and South America specimens only] [2].―Mantelatto and Fransozo, 2000: 703 [8].―Bertini *et al*., 2004: 2204 [120].―Ariza *et al*., 2008: 157, 163–164, fig. 3 [130].―Hirose *et al*., 2012: 17–30 [9].

Material examined.―Photograph of holotype (by H. Taylor).―Locality not given, 1 ♀ of *Persephona lichtensteinii* (CW 26.0mm), NHMUK White 1 97.b.―Photograph of paratype (by H. Taylor).―Locality not given, 1 ♂ of *Persephona lichtensteinii* (CW 27,2mm), NHMUK White 1 97.a.―Holotype.―São Sebastião, Brazil, id: M. J. Rathbun, 1 ♂ of *P*. *finneganae* (CW 36.5mm), USNM 67989.―Paratype.―São Sebastião, Brazil, id: M. J. Rathbun, 1 ♂ of *P*. *finneganae*, MZUSP 934. Other specimens.―Honduras: Gracias a Dios, Punta Patuca ULLZ 2002 (1 ♂). Colombia: Ceycen Island USNM 1072260 (1 ♂). Venezuela: USNM 1155107 (1 ♂ reported as *P*. *finneganae*), 1155108 (1 ♂ reported as *P*. *finneganae*). Brazil: Amapá MNRJ 328 (2 ♂ reported as *P*. *finneganae*); MZUSP 8575 (1 ♀ov); MNRJ 1456 (1 ♂, 1 ♀ reported as *P*. *crinita*). Alagoas, Maceió MZUSP 6789 (2 ♂, 2 ♀ov), 6893 (1 ♂), 21096 (1 ♂); MZUSP 21087 (1 ♀ov reported as *P*. *crinita*). Sergipe, Pirambu MZUSP 6616 (1 ♂). Bahia, Ilhéus MZUESC 298 (6 ♂, 4 ♀); MZUSP 21088 (1 ♀ov reported as *P*. *crinita*), 21090 (1 ♂ reported as *P*. *crinita*), 21091 (2 ♂ reported as *P*. *crinita*). Espírito Santo, Guarapari MZUSP 12160 (4 ♂, 8 ♀). Cachoeira do Itapemirim MNRJ 733 (2 ♂, 1 ♀ reported as *P*. *finneganae*). Regência MNRJ 16163 (1 juvenile reported as *P*. *crinita*). Rio de Janeiro, São Francisco de Itabapoana MNRJ 326 (4 ♂, 4 ♀ reported as *P*. *finneganae*), 1460 (2 ♂, 2 ♀). São João da Barra MNRJ 3894 (2 ♂, 4 ♀). Sepetiba MZUSP 8372 (1 ♀ov). Ilha Grande MZUSP 2326 (1 ♀ov). São Paulo, Caraguatatuba CCDB 759 (1 ♀ reported as *P*. *crinita*). Ubatuba CCDB 0019 (1 ♂), 0023 (1 ♂ reported as *P*. *crinita*), 1430 (2 ♂, 1 ♀), 2857 (1 ♀ reported as *P*. *crinita*), 0011 (1 ♂ reported as *P*. *crinita*), 0019 (1 ♂ reported as *P*. *crinita*). São Sebastião MNRJ 3897 (2 ♂). Guarujá MZUSP 3370 (1 ♀). Santa Catarina MZUSP 13102 (1 ♂, 1 ♀). São Francisco do Sul MNRJ 1457 (1 ♂ reported as *P*. *crinita*).

Revised diagnosis.―Carapace with seven marginal spines or tubercles, three long spines or tubercles on posterior margin, two spines on lateral margin, two spines or tubercles on subhepatic margin; adult background carapace color pale rose pink to bluish off-white, normally uniform darker rose area dominating most of dorsal surface, often with diffuse withe color at the spines, without symmetrical dark patterning of lines or spots; front not prominent, width usually more than three times length. Cheliped propodus length usually exceeding 4 ½ times width, carpus and distal merus without tufts of setae on inner margins. Go1 distal opening narrow, directed towards ventral face; apex simple, without prominent lobe on mesiodistal margin, with subapical setae (Fig 7C).

Distribution.―Western Atlantic: Caribbean Sea, Honduras, Colombia, Venezuela, Suriname, French Guiana and Brazil (from Amapá to Santa Catarina).

Remarks: The holotype and paratype photographs of *P*. *lichtensteinii* (NHMUK White 1 97.b and NHMUK White 1 97.a, respectively) were analyzed, as well as the holotype and paratype of *P*. *finneganae* (USNM 67989 and MZUSP 934, respectively). Based on original description of Leach [76] we are sure about the identity of *P*. *lichtensteinii* and the synonym of *P*. *finneganae* to this.

***Persephona mediterranea*** (Herbst, 1794)

Figs 6D–6F and 8D–8F

*Cangrejo Tortuga*.―Parra, 1787: 137, pl. 51, fig. 2 [131].

*Cancer mediterraneus* Herbst, 1794: 150, tab. 37, fig. 2 [67].

*Leucosia mediterranea* Lichtenstein, 1815: 142 [132].

*Guaia punctata*.―H. Milne-Edwards, 1837: 127 [part] [77].

*Persephona latreillei* Leach, 1817: 22 [76]

*Persephona guaia* Bell, 1855: 292 [18]

*Persephone punctata* [sic].―von Martens, 1872: 113 [133].―Moreira, 1901: 35 [134].―Luederwaldt, 1919: 435 [126].

*Persephona punctata*.―Miers, 1886: 312, pl. 25, fig. 5 [135].―Rodriguez, 1980: 254, lam. 12 [94].―Takeda, 1983: 116 (unnumbered photograph). [116].―Tavares, 1993: 18, 21, pl. 5, fig. F. [part] [136].

*Persephona punctata punctata*.―Rathbun, 1937: 152–154, pl. 42, fig. 6,7 [part] [3].―Lemos de Castro, 1962: 38, est. 1, figs. 3–4 [137].

*Persephona punctata aquilonaris*.―Holthuis, 1959: 183 [6].―Abreu, 1980: 3 [138].

*Persephona aquilonaris*.―Guinot-Dumortier, 1959: 429–433, fig. 7, 9 [part, Caribbean and South America specimens only] [12].―Fausto-Filho, 1968: 44 [139].―Coelho and Ramos, 1972: 183 [113].

*Persephona mediterranea*.―Abele, 1970: 62–63 [part, not full Gulf of Mexico range] [91].―Powers, 1977: 39 (part, not full Gulf of Mexico range) [16].―Coelho and Torres, 1980: 72 [129].―Coelho *et al*., 1986: 100 [117].―Bordin, 1987: 9 [140].―Negreiros-Fransozo *et al*., 1989: 177 [141].―Raz-Guzman and Sánchez, 1992: 30–31, pl. I-1[14].―Melo, 1996: 153 (unnumbered figure, map) (part, not full Gulf of Mexico range) [2].―Souza, 1997: 38 [142].―Torres, 1998: 115–120, fig. 29 [7].―Boschi, 2000: 112 [98].―Mantelatto and Fransozo, 2000: 702–703, 705, 707 [8].―Mantelatto *et al*., 2003: 211–212 [119].―Branco and Fracasso, 2004: 299 [143].―Braga *et al*., 2005: 3, 28, 32, fig. 9 [144].―Coelho *et al*., 2008: 15 [122].―Ng *et al*., 2008: 93 [1].―Martínez *et al*., 2009: 279 [145].―Bertini *et al*., 2010: 7 [146].―Carvalho *et al*., 2010: 109 [123].―Vieira and Calazans, 2010: 432 [147].―Hirose *et al*., 2012: 17–30 [9].

Material examined.―Photograph of lectotype (by C. O. Coleman).―Mediterranean Sea (error), 1 ♂ of *Cancer mediterraneus* (CW 29.0mm), ZMB 0756.―Photograph of type (by H. Taylor).―West Indies, 1 ♀ of *Persephona latreillei* (CW 42.0mm), NHMUK White 1 96.d (Sloane 2048).―Photograph of syntype (by M. Carnall).―Antilles islands, 1 ♂ of *Persephona guaia* (CW 45.0mm), OUMNH 13775. Other specimens.―United States: Florida, Tampa UFMNH 6579 (1 ♀). Belize: Twin Cays ULLZ 15385 (1 juvenile). Brazil: Espírito Santo MNRJ 23309 (1 ♀ov reported as *P*. *punctata punctata*). Rio de Janeiro, Cabo Frio MNRJ 3900 (1 ♂, 1 ♀). Arraial do Cabo MNRJ 3899 (1 juvenile reported as *P*. *punctata punctata*). Rio de Janeiro MNRJ 333 (3 ♂ reported as *P*. *punctata punctata*), 334 (2 ♀ reported as *P*. *punctata punctata*), 346 (1 ♂, 1 ♀ov reported as *P*. *punctata punctata*), 712 (1 ♂ reported as *P*. *punctata punctata*). Guaratiba MNRJ 342 (1 ♂ reported as *P*. *punctata punctata*). Angra dos Reis MNRJ 3897 (1 ♂ reported as *P*. *punctata punctata*), 3898 (1 ♂, 3 ♀), 4462 (1 ♂). Ilha Grande MNRJ 4463 (3 ♂, 2 ♀ reported as *P*. *punctata punctata*). São Paulo, Cananéia MZUSP 33205 (1 ♂). Caraguatatuba CCDB 758 (1 ♂). Ubatuba CCDB 1539 (2 ♀, 2 juveniles), 2836 (1 ♂), 3881 (1 ♂). Santos MNRJ 23308 (2 ♂, 1 ♀ reported as *P*. *punctata punctata*). Santa Catarina, São Francisco do Sul CEPESUL 113 (1 ♂).

Revised diagnosis.―Carapace with three marginal spines, all posterior; adult background carapace color beige to off-white, typically marked by 7–14 large reddish brown to light orange or tan spots distributed symmetrically to each side of light unspotted median line, each large spot usually margined by narrow darker line encircling multiple granules, dark spots separated by broad lighter veins of background color, veins and rows of large spots not obviously converging posteriorly nor broken into small spots posterolaterally; front prominent, width usually two and half times length. Cheliped with propodus usually more than 3 times longer than wide, carpus and distal portion of merus with tufts of setae on inner margins. Go1 with distal opening large, directed towards mesial face; apex simple, with prominent lobe on mesiodistal margin, without subapical setae at the distal margin (Fig 8D–8F).

Distribution.―Western Atlantic: Eastern Gulf of Mexico, Caribbean Sea, Antilles, Venezuela, Suriname, French Guiana, Brazil to Uruguay.

Remarks.―Bell ([18]: 292–293) analyzed the types specimens of Leach at the British Museum and concluded that *P*. *lamarckii* and *P*. *latreillei* were identical, considering the differences on the pterygostomian region as variation. He noted that the coloration of those specimens were not preserved, not being possible the identification of theses specimens as *P*. *mediterranea* (*Cancer mediterraneus*) by Herbst. He also commented that Browne does not mentioned coloration on his description to *Cancer punctatus*. Thus, Bell synonymizes both Leach species to *Persephona guaia*. Rathbun ([3]: 153) considered the three species as synonyms of the *Persephona punctata punctata*, and those animals with occurrence from West Indies to Brazil. The specimens from United States, from New Jersey to Texas, should be considered *Persephona punctata aquilonaris*, as stated on introduction. We analyses the photographs of the types of *P*. *lamarckii* (NHMUK White 1 96a), *P*. *latreillei* (NHMUK White 1 96d), *P*. *guaia* (OUMNH 13775), and the specimen of *P*. *puntata aquilonaris* (USNM 62057). Based on morphology, we could infer that *P*. *lamarckii* is clearly a junior synonym of *P*. *punctata* with acute margin on the pterygostomian region and front not prominent. While *P*. *latreillei* might be a junior synonym of *P*. *mediterranea*. The specimen has obtuse margin and prominent front, from West Indies. Although we do not have access to DNA, color pattern or gonopod morphology, we could infer based on Rathbun’s and our pattern of distribution of the species *P*. *mediterranea* and *P*. *aquilonaris*, that the specimens from Caribbean to Brazil are indeed *P*. *mediterranea* and those from New Jersey to Gulf of Mexico are *P*. *aquilonaris*. According to DiMauro ([106]: 173) the specimen *P*. *guaia* (OUMNH 13775) is registered as syntype of Bell, from West Indies. Based on the photography, we could identify the same pattern of coloration of *P*. *mediterranea* (*Cancer mediterraneus*) by Herbst, and the obtuse margin on pterygostomian region. Thus, we concluded that *P*. *guaia* by Bell is in fact a junior synonym of *P*. *mediterranea*. With the analysis of the specimen of *P*. *puntata aquilonaris* (USNM 62057) from St. Augustine, Florida we could identify obtuse margin and prominent front, but also the different pattern of coloration on carapace, as described in *P*. *aquilonaris* by Rathbun ([3]: 154). Thus, the holotype of *P*. *punctata aquilonaris* is in fact correctly as synonym of *P*. *aquilonaris*.

***Persephona orbicularis*** Bell, 1855

Fig 7D and 7G

*Persephona orbicularis* Bell, 1855: 294, pl. 31, fig.7 [18].―Rathbun, 1937: 160, pl. 45, figs. 5–6 [3].―Garth, 1957: 15 [17].―Boschi, 2000: 112 [98].―Wehrtmann and Echeverría-Sáenz, 2007: 124 [20].―Ng *et al*., 2008: 93 [1].―Vargas-Castilho, 2008: 108 [107].―Moscoso, 2012: 19 [111].

*Myra townsendi* Rathbun, 1893: 255 [148].

*Persephona townsendi*.―Rathbun, 1898: 613 [149].―1910: 594.―Rathbun, 1937: 160, pl. 42, fig. 1, pl. 43, fig. 1 [3].―Crane, 1937: 104 [150].―Garth, 1948: 18 [151].―Garth, 1960: 121 [101].―Garth, 1966: 9 [99].―Buitendijk, 1950: 271 [103].―Paul and Hendrickx, 1980: 10 [152].―DiMauro, 1982: 174 [106].―Rodríguez de la Cruz, 1987: 119 [153].―Correa-Sandoval, 1991: 13 [107].―Lemaitre and Alvarez-León, 1991: 51 [154].―Hendrickx, 1993: 8 [108].―Hendrickx, 1995: 129 [109].―Hendrickx, 1996: 616 [155].―Hendrickx, 1997: 150, fig. 106 [19].―Boschi, 2000: 113 [98].―Ng *et al*., 2008: 93 [1].―Moscoso, 2012: 19 [111].

Material examined.―Photograph of holotype (by M. Carnall).―Valparaiso, Chile, 1 ♀ of *P*. *orbicularis* (CW 38.1mm), OUMNH 13776.―Holotype.―Gulf of California, Mexico, 1♂ of *P*. *townsendi* (CW 31.7mm), USNM 17382. Other specimens.―Sinaloa, Mexico: CCDB 3025 (1 ♀ reported as *P*. *townsendi*). Costa Rica: CCDB 2939 (3 ♂, 1 ♀, 1 juvenile). Panamá: ULLZ 10078 (1 juvenile); ULLZ 13931 (2 juveniles reported as *P*. *townsendi*).

Revised diagnosis.―Carapace with five marginal spines or tubercles, three long spines on posterior margin, two spines at subhepatic margin; adult background carapace color pale dark reddish to purplish, sometimes with defined slightly darker reddish patterning of veins distributed asymmetrically to each side of orange light spotted median line, area dominating most central part of the carapace; front not prominent, usually width more than three times length. Chelipeds propodus length usually more than four times width, carpus and distal part of merus without tufts of setae on inner margins. Go1 distal opening narrow, directed towards meisoventral face; apex simple, without prominent lobe on mesiodistal margin, with subapical setae (Fig 7D and 7G).

Distribution.―Pacific Oriental: Mexico (Punta San Fermin, Gulf of California to Oaxaca), Costa Rica, Panama, Colombia, Ecuador, and Peru.

Remarks.―The type locality of *P*. *orbicularis* reported as Valparaiso, Chile by Bell [18]. The type specimen was brought by Mr. Miller, a surgeon in the British Royal Navy, and deposited into the Bell collection [18]. However, Boone [71] questioned the occurrence of *P*. *orbicularis* in Chile. Garth [17] also suggested that this species was never found in Chile. He argued that species of *Persephona* have a tropical distribution in the Americas tropics; thus *P*. *orbicularis* may have been collected farther north than Chile. Nonetheless, he suggested that the occurrence of *P*. *orbicularis* collected by Boone (Panama) it is within the range of distribution of *P*. *townsendi*, which could indicate that the last is synonymous of the first. Based on morphological and molecular analyses we conclude that the *P*. *townsendi* is a junior synonymy of *P*. *orbicularis*. The distribution of *P*. *orbicularis* goes from the Gulf of California to Peru. This represents another mistake in the type locality as it was observed for *P*. *mediterranea* (see remarks).

***Persephona punctata*** (Linnaeus, 1758)

Figs 5C and 7E

*Cancer* (three thorned crab) Browne, 1756:422 [156], pl. 42, fig. 3.

*Cancer punctatus* Linnaeus, 1758: 630 [78].―Herbst, 1794: 89 [67], pl. 11, figs. 15, 16.

*Persephona lamarckii* Leach, 1817: 23 [76].

*Guaia punctata*.―H. Milne Edwards, 1837: 127 [part] [77].―Gibbes, 1850: 185 [11].―Desbonne, 1867: 53 [157].

*Persephona punctata*.―Stimpson, 1859: 70 [10].―Rathbun, 1901: 87 [158].―Rathbun, 1933: 98, fig. 94 [62].―Boone, 1930: 54, pl. 10, fig. B [73].―Guinot-Dumortier, 1959: 428, figs. 5 a,b, 6 [12].―Holthuis, 1959: 183 [6].―Fausto-Filho, 1966: 33 [159].―Coelho and Ramos, 1972: 183 [113].―Gomes-Corrêa and Silva-Brum, 1980: 61 [114].―Rodriguez, 1980: 254, pl. 12 [94].―Coelho *et al*., 1983: 154 [115].―Coelho *et al*., 1986: 100 [117].―Taissoun, 1986–88: 130, 131 (figure) [87].―Bordin, 1987: 9 [140].―Werding and Müller, 1989: 410 [128].―Tavares, 1993: 18, 21, pl. 5, fig. F. [part.] [136].―Melo, 1996: 154 (unnumbered figure, map) [2].―Torres, 1998: 126, fig. 31 [7].―Boschi, 2000:112 [98].―Mantelatto and Fransozo, 2000: 702–703, 705, 707 [8].―Branco and Fracasso, 2004: 299 [143].―Braga *et al*., 2005: 3, fig. 9 [144].―Ng *et al*., 2008: 93 [1].―Martínez *et al*., 2009: 279 [145].―Carvalho *et al*., 2010: 109–113 [123].―Hirose *et al*., 2012: 17–30 [9].

*Persephone punctata* [sic].―Luederwaldt, 1919: 435 [126].

*Persephona punctata punctata*.―Rathbun, 1937: 152, pl. 42, figs. 2–3 [3].―Rodrigues da Costa, 1968: 334 [160].

*Persephona mediterranea*.―Martínez-Iglesias and Gómez, 1986: 11, fig. 6 a [161].

Material examined.―Photograph of holotype (by H. Taylor).―Locality not given, 1 ♂ of *Persephona lamarckii* (CW 52.0mm), NHMUK White 1 96.a.―Neotype.―Costa Rica, Isla Pajaros, Limón, 1 ♂ of *Persephona punctata* (CW 29.88mm), MZUCR 2470–4. Other specimens.―Suriname: Saramacca USNM 103479 (1 ♂, 1 ♀). Brazil: Pará MNRJ 348 (1 ♀ reported as *P*. *punctata punctata*). Ceará, Fortaleza MNRJ 332 (08 ♂, 10 ♀, 2 ♀ov, 4 juveniles reported as *P*. *punctata punctata*), 350 (2 juveniles reported as *P*. *punctata punctata*). Bahia, Itacaré MNRJ 2460 (2 ♂, 2 ♀, 3 ♀ov, 4 juveniles reported as *P*. *punctata punctata*). Cumuruxatiba MNRJ 14424 (1 ♂). Espírito Santo, Linhares MNRJ 339 (1 ♂, 1 ♀ov female reported as *P*. *punctata punctata*). Guarapari MNRJ 4464 (4 ♂, 4 ♀ov), 4465 (2 ♂, 2 ♀ov). Anchieta MNRJ 4461 (1 ♂, 2 ♀ov, 1 juvenile). Rio de Janeiro, São Francisco de Itabapoana MNRJ 344 (1 ♂ reported as *P*. *punctata punctata*), 13754 (1 juvenile). Rio de Janeiro MNRJ 347 (1 juvenile reported as *P*. *punctata punctata*). São Paulo, Ubatuba CCDB 0022 (1 ♀ov), 0027 (1 ♂), 2529 (1 ♂), 2856 (2 ♂, 2 ♀), 3049 (1 ♂). Caraguatatuba CCDB 0755 (1 ♀), 1532 (3 juveniles). São Vicente MNRJ 335 (1 ♂ reported as *P*. *punctata punctata*). Santos MNRJ 331 (1 ♂ reported as *P*. *punctata punctata*), 343 (2 ♂, 1 ♂).

Revised diagnosis.―Carapace with five marginal spines or tubercles, 3 long spines on posterior margin, 2 tubercles at subhepatic margin; adult background carapace color pale purplish to bluish off-white, sometimes with defined slightly darker purplish patterning of anastomosed spots distributed symmetrically to each side of orange light spotted median line, dark spots separated by narrow lighter veins of background color, veins and lines of intersecting spots converging somewhat posteriorly, area dominating most of frontal dorsal surface, but diffused purple color, often with diffuse pale brown to orange near frontal margin and subhepatic margin; front not prominent, width usually four and half times length. Chelipeds with propodus usually more than 3 times and half times longer than wide, carpus and distal part of merus with tufts of setae on inner margins. Go1 with distal opening wide, directed towards ventral face; apex expanded laterally, without prominent lobe on mesiodistal margin, without subapical setae (Fig 7E).

Distribution.―Western Atlantic: Caribbean Sea, Antilles, Colombia, Venezuela, Guianas, Suriname, and Brazil (from Amapá to Rio Grande do Sul).

Remarks.―Linnaeus ([78]: 630) described *Cancer punctatus*, based on Rumphius (Asian iconotype) and a Browne (Jamaican iconotype) plates, with the following characters: “brachyurus, thorace obovato punctato, postice tridentato”. However, the Rumphius animal was identified by Fabricius ([162]: 350, 351) as *Leucosia fugax* (*Myra fugax* (Fabricius)) and the American animal as *Leucosia punctata* Bell, 1855. Holthuis ([6]: 183) selected the specimen figured by Browne ([156]: 422, pl. 42 fig. 3) as the lectotype of *Cancer punctatus* Linnaeus, 1758. Thus, the type locality for the species is at present Jamaica, British West Indies. Nevertheless, the type specimen of Linnaeus is not extant [1] and the iconotype selected by Holthuis [6] is a very poorly detailed drawing. Then, we must select another type. We suggest the election of a specimen analyzed by us, morphological and molecularly. We select a male from Costa Rica, Isla Pajaros, Limón (MZUCR 2470–4) as neotype of *Cancer punctatus* Linnaeus, 1758, although we understand that a specimen from Jamaica maybe preferable. The type locality becomes Costa Rica.

***Persephona subovata*** (Rathbun, 1894)

Fig 7F

*Myra subovata* Rathbun, 1893: 256 [148].

*Persephona subovata*.―Rathbun, 1898: 613 [149].―Rathbun, 1937: 158, pl. 43, figs. 4–5 [3].―Hendrickx, 1995: 129 [109].―Hendrickx, 1997: 149 [19].―Boschi, 2000: 112 [98].―Ng *et al*. 2008: 93 [1].―Vargas-Castilho, 2008: 108 [110].

*Persephona edwardsii* [error].―Boone, 1930: 53, pl. 10, fig. A [74].

*Iliacantha hancocki* Rathbun, 1935: 2 [163].―Maurer *et al*. 1984: 51 [164].―Ng *et al*. 2008: 91 [1].―Vargas-Castilho, 2008: 108 [110].

Material examined.―Holotype.―South of Tiburon Island, Sonora, Mexico, 1 ♀ of *P*. *subovata* (CW 20.2mm), 1 juvenil (CW 12.3mm), USMN 17385.―Holotype.―Santa Maria Bay, Lower California, Mexico, 1 ♂ of *I*. *hancocki* (CW 20.3mm), USMN 69260. Other specimens.―Mexico: Oaxaca CNCR 3269 (2 ♂, 1 ♀). Costa Rica: CCDB 1644 (2 ♀) 2834 (1 ♂) (reporter as *I*. *hancocki*).

Revised diagnosis.―Carapace with three marginal spines or tubercles, three long spines on posterior margin; adult background carapace color reddish or orange, with darker red area dominating most of central and frontal dorsal surface, without symmetrical dark patterning of lines or spots; front slightly produced, width usually two times length. Cheliped propodus length usually more than six times width, carpus and distal part of merus without tufts of setae on inner margins. Go1 distal opening narrow, directed towards meisodorsal face; apex simple, without prominent lobe on mesiodistal margin, with subapical setae (Fig 7F).

Distribution.―Pacific Oriental: Mexico (Punta Abreojos, Lower California South (West coast), Angel de la Guarda Island and Tiburon Island, Sonora (East coast)), Costa Rica, Panama, Colombia, Ecuador, and Peru.
